# Use of a head-to-tail peptide cyclase to prepare hybrid RiPPs†

**DOI:** 10.1039/d3cc04919a

**Published:** 2024-06-04

**Authors:** Tung Le, Dongtianyu Zhang, Rachel M. Martini, Subhanip Biswas, Wilfred A. van der Donk

**Affiliations:** a Department of Chemistry and Howard Hughes Medical Institute, University of Illinois at Urbana-Champaign Urbana IL 61801 USA vddonk@illinois.edu; b Department of Biochemistry, University of Illinois at Urbana-Champaign Urbana IL 61801 USA

## Abstract

Cyclotides and lanthipeptides are cyclic peptide natural products with promising bioengineering potential. No peptides have been isolated that contain both structural motifs defining these two families, an N-to-C cyclised backbone and lanthionine linkages. We combined their biosynthetic machineries to produce hybrid structures that possess improved activity or stability, demonstrate how the AEP-1 plant cyclase can be utilised to complete the maturation of the sactipeptide subtilosin A, and present head-to-tail cyclisation of the glycocin sublancin. These studies show the plasticity of AEP-1 and its utilisation alongside other post-translational modifications.

## Abstract 2

The biosynthetic enzymes that install the characteristic features of cyclotides and lanthipeptides were combined to make new-to-nature compounds.

## Graphical abstract

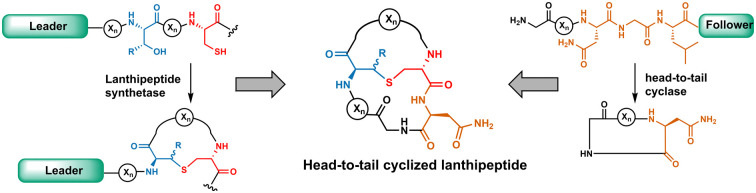

With over 40 drugs currently in clinical use and one new drug entering the market every year, cyclic peptides are among the most promising chemical modalities for next-generation drug discovery.^1^ Favourable properties such as constrained conformational flexibility combined with large surface area provide cyclic peptides with improved stability and binding affinity for targets that are challenging for small molecule drugs, including protein–protein interactions.^2,3^ Given these favourable features, it is not surprising that diverse strategies evolved in nature to access these scaffolds, among which lanthipeptides and cyclotides feature as prominent examples from the family of ribosomally synthesised and post-translationally modified (PTM) peptides (RiPPs).

The biosynthesis of most RiPPs commences with translated precursor peptides that contain N-terminal leader sequences followed by C-terminal core peptides.^4^ Biosynthetic enzymes engage the leader sequences and catalyse class-specific modifications on the core peptides. In the case of lanthipeptides (Fig. 1a), the PTMs involve dehydration of Ser/Thr residues to generate dehydroalanine (Dha)/dehydrobutyrine (Dhb), followed by intramolecular Michael-type addition of Cys thiols to the dehydrated residues to form the class-defining lanthionine (Lan) and methyllanthionine (MeLan) linkages.^5^

**Fig. 1:**
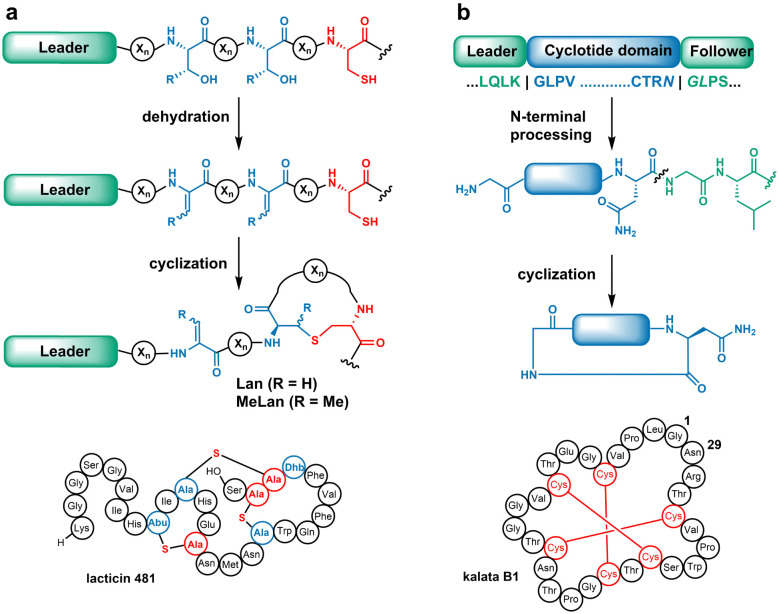
(a) General biosynthesis scheme for lanthipeptides and structure of a representative lanthipeptide lacticin 481 (b) general biosynthesis scheme and structure of the cyclotide kalata B1. The recognition motif for OaAEP is italicised.

When multiple pairs of cognate residues are present in the precursors (*e.g.*Fig. 1a), highly cross-linked ring patterns result.^5^ These thioether bridges endow desirable properties including antiviral, antifungal, and antimicrobial activities.^6^

Cyclotides are plant-derived macrocyclic peptides typically containing cystine knots and a head-to-tail cyclic backbone (Fig. 1b). Cyclotide biosynthesis involves a precursor peptide with a core domain flanked by N- and C-terminal recognition sequences. For example, enzymatic removal of the N-terminal peptide by a papain-like cysteine protease,^7^ followed by head-to-tail cyclisation catalysed by an asparaginyl endopeptidase from *Oldenlandia affinis* (OaAEP1), results in the mature product kalata B1.^8^ Macrocyclization renders cyclotides with exceptional stability and bioactivity.^9^

Both lanthipeptide synthetases and the OaAEP1 cyclase have been shown to be forgiving with regards to substitutions in the core peptide sequences, accelerating the study of structure–activity relationships^10–14^ and bioengineering efforts.^13,15–21^ As the PTMs of these natural products are orthogonal in terms of cognate residues and mode of cyclisation, we sought to combine the merits of both systems to produce hybrid peptides that contain both lanthionine linkages and a head-to-tail cyclised structure. We show such hybrid structures are attainable and can impart changes in bioactivity or stability. We also show that the head-to-tail cyclic sactipeptide subtilosin A can be produced using OaAEP1 and that a member of another RiPP class, the glycocin sublancin, can be cyclised.

*Preparation of subtilosin A and cyclised sublancin using OaAEP1*. Subtilosin A is produced by *Bacillus subtilis* and harbors bioactivity against a range of bacteria.^22,23^ Its structure features thioether crosslinks involving the sulfur atoms on Cys and the Cα of cognate residues, together with a head-to-tail cyclic backbone (Fig. 2a).^24^ The thioether bonds are installed in the precursor peptide SboA by the radical SAM enzyme AlbA^25^ and heterologous generation of the thioether linkages in *Escherichia coli* has been achieved.^26^ However, the head-to-tail cyclisation, proposed to be catalysed by a protease encoded in the subtilosin biosynthetic gene cluster,^27,28^ has not been reconstituted. Thus, we sought to achieve this final maturation step by OaAEP1-mediated cyclisation (Fig. 2a). Thioether linkages were installed first by co-expression of His-tagged SboA containing an engineered C-terminal AsnGlyLeu (NGL) motif with the synthetase AlbA together with the pACYC-sufABCDSE plasmid containing *E. coli* genes for iron–sulfur cluster assembly (Fig. 2a; step 1).^29^ Lys-N protease was then used to cleave the leader peptide including Asn1 of the core peptide, yielding peptide SboA(NGL)(-N1) (Fig. 2a, step 2). Finally, treatment with a previously described OaAEP1 variant that improves cyclisation at the NGL motif^30^ resulted in loss of Gly-Leu dipeptide and a water molecule, achieving head-to-tail cyclisation and replacing the lost Asn from the previous proteolytic step with the Asn residue near the C-terminus (Fig. 2a, step 3). LC-MS analysis revealed that two isomers with the mass of cyclised subtilosin were produced, one of which matched the retention time of native subtilosin A (Fig. S1a, ESI†). MS/MS analysis of both isomers and the standard did not result in any b/y ions, suggesting they were head-to-tail cyclised (Fig. S1b, ESI†). We hypothesise that the second product involves a lactam with the side-chain amine of Lys1 in SboA(NGL)(-N1), resulting in an isopeptide bond to the C-terminus. This assignment is supported by observed protection from Lys-C digestion (Fig. S1c, ESI†) and by the observed reactivity with 2-pyridine carboxaldehyde (Fig. S1d, ESI†), a reagent that is selective for N-terminal amino groups in peptides.^31^ Presumably, the constraints induced by the thioether linkages (Fig. S1e, ESI†) makes it more difficult for the N-terminal amine to cyclise resulting in competing cyclisation involving the side chain amine of Lys1. Amine nucleophiles other than N-terminal amino groups have been documented previously to be substrates for OaAEP1.^32^ We also observed a side-product resulting from a competing hydrolysis reaction (Fig. 2b), which fragmented into b/y ions characteristic of non-N-to-C cyclised sactipeptides (Fig. S2, ESI†) under similar conditions used to test the cyclised compounds. Since commercial Lys-N protease is no longer available, we also created mutant SboA(NGL)(insR2) where we inserted an Arg residue N-terminal to Lys2, followed by endoproteinase ArgC cleavage to again yield SboA(NGL)(-N1) (ESI†). Using a similar approach, we also N-to-C cyclised the glycocin sublancin (Fig. S3, ESI†) resulting in product with comparable antimicrobial activity as the wild-type compound (Fig. S4, ESI†).

**Fig. 2:**
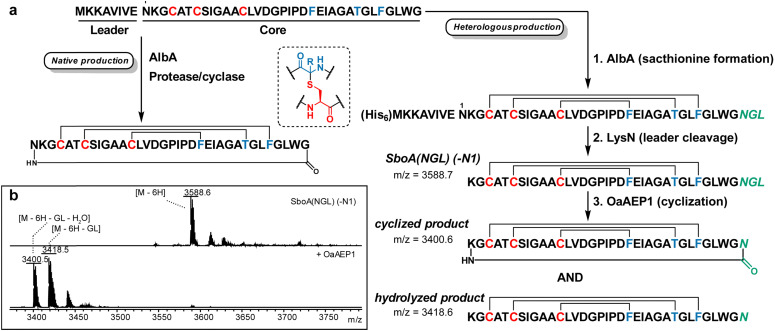
(a) Production of subtilosin A from precursor peptide SboA. The structure of the sactionine linkage is shown in the dashed box. In the precursor peptide sequence, a His-tag and NGL motif for OaAEP1 cyclization was added. (b) MALDI-TOF MS result of (top) LysN-digestion of SboA(NGL) after co-expression with AlbA and (bottom) OaAEP1 cyclization of the purified product from the LysN digestion.

*Head-to-tail cyclised lanthipeptides using OaAEP1*. We next investigated several lanthipeptides based on the prochlorosin 2.8 scaffold as substrates for OaAEP1-catalysed cyclisation. Prochlorosins are made by a class II lanthipeptide synthetase ProcM that installs lanthionine linkages on a wide range of precursor peptides.^33,34^ Previous studies exploited ProcM's remarkable substrate tolerance for library generation^19,21^ and epitope grafting.^35^ We wondered if further conformational restriction *via* head-to-tail cyclisation would improve binding affinity of the hits from these latter studies.

Lanthipeptide precursor genes were cloned to encode an N-terminal His-tag and C-terminal NGL motif, and inserted into a co-expression construct with *procM*. After expression in *E. coli*, metal affinity purification, and leader peptide removal using the substrate tolerant protease LahT150,^36^ the modified core peptides were used for OaAEP1 cyclisation. We selected two engineered prochlorosin 2.8 variants that bind αvβ3 integrin in the micromolar and nanomolar range,^35^ 15RGD and 16RGD, as candidates. Matrix-assisted laser desorption ionisation time-of-flight mass spectrometry (MALDI-TOF MS) analysis demonstrated these peptides were fully modified by ProcM (Fig. 3a and c; Fig. S6, ESI†). Upon treatment with OaAEP1, both modified core peptides were cyclised, indicated by a mass loss of 188 Da (Fig. 3b and d). We did not observe side-products resulting from hydrolysis at the NGL motif (mass loss of 170 Da).

**Fig. 3:**
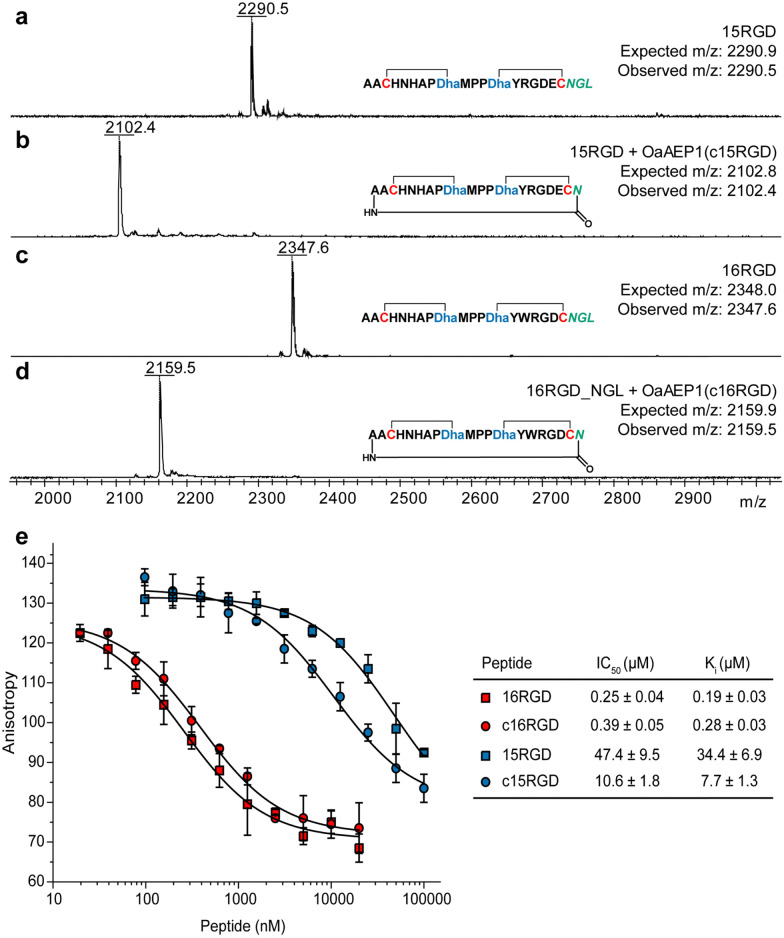
(a)–(d) OaAEP1-catalysed head-to-tail cyclisation of prochlorosin 15RGD and 16RGD. (e) Competition fluorescence polarisation curves for 15RGD, 16RGD and their N-to-C cyclised variants (for ESI MS data, see Fig. S5 and S6, ESI†).

*Protease resistance and binding affinity*. The purified OaAEP1-cyclized lanthipeptides, c15RGD and c16RGD, were challenged with aminopeptidase. Compared to the uncyclised compounds, the circular peptides were resistant to aminopeptidase (Fig. S7, ESI†). We then assessed their affinity for αvβ3 integrin *via* fluorescence polarisation competition against fluorescently labelled c(RGDyK), a known integrin binder.^35,37^ c16RGD showed slightly reduced affinity, from *K*_i_ = 0.19 ± 0.03 μM (noncyclised) to 0.28 ± 0.03 μM (N-to-C cyclised), whereas c15RGD showed 4-fold higher affinity, from *K*_i_ = 34.4 ± 6.9 μM (noncyclised) to 7.7 ± 1.3 μM (Fig. 3e).

*Head-to-tail cyclised lacticin 481*. Next, we cyclised lacticin 481 (Fig. 1a), a lanthipeptide that is active against a range of Gram positive bacteria.^38^ A similar strategy was adapted to that described above. His-tagged LctA_NGL was co-expressed with the lanthipeptide synthetase LctM, purified and cleaved with trypsin to yield the modified core peptide (Fig. 1a) without Lys1, which was previously shown to be non-essential for bioactivity.^39^ Subsequent OaAEP1 treatment resulted in two core peptides, which were separated by RP-HPLC, one of which was N-to-C cyclised by OaAEP1 while the other was hydrolysed by OaAEP1 (Fig. S8a–d, ESI†). While the non-cyclised lacticin 481_NGL variant displayed antimicrobial activity against *Lactococcus lactis* sp. cremoris, suggesting correctly installed thioether rings, the cyclised product did not display such activity (Fig. S8e, ESI†) showing that N-to-C cyclisation can be deleterious.

RiPPs have been attractive targets for generating new-to-nature designer peptides.^40,41^ These studies have led to hybrid structures with motifs originating from different RiPP classes. In almost all cases, hybrid RiPPs were accessed by engineering leader peptide chimeras,^42^ by swapping leader peptides,^43^ or by using enzymes that do not need recognition sequences or use common recognition sequences.^41,44–46^ In this study, we complement these prior studies by using an N-terminal leader sequence for installation of crosslinks between side chains by enzymes from various bacterial RiPP classes and a C-terminal recognition sequence for N-to-C cyclisation by an enzyme from another, plant-derived RiPP class. The latter recognition sequence was added to the C-terminus of precursor peptides of lanthipeptides, sactipeptides and glycocins. We show that OaAEP1 can cyclise a range of RiPPs with different ring patterns into head-to-tail cyclic compounds. To the best of our knowledge, there is no precedence for such engineered hybrid structures. We anticipate that the methods in this study as well as the use of alternative head-to-tail cyclisation enzymes^47–50^ or in cellulo strategies^51^ could be applied to other RiPPs as a general tool to enhance peptide stability and/or bioactivity.

T. L.: conceptualisation, investigation, writing. D. Z., R. M., S. B. investigation; W. A. V.: conceptualisation, supervision, writing.

This work was supported by the National Institutes of Health (R37 GM058822). WAV is an Investigator of the Howard Hughes Medical Institute and Partner Investigator of the Australian Centre of Excellence for Innovations in Peptide and Protein Science.

## Conflicts of interest

There are no conflicts to declare.

## Supplementary Material

CC-060-D3CC04919A-s001
