# Supplementary material for: Use of a head-to-tail peptide cyclase to prepare hybrid RiPPs
Source: Chem Commun (Camb). 2024 Jun 4;60(51):6508–11. doi: 10.1039/d3cc04919a (PMC11189026; doi:10.1039/d3cc04919a)
Supplement: CC-060-D3CC04919A-s001 [file CC-060-D3CC04919A-s001.pdf]

Electronic Supplementary Material (ESI) for ChemComm.
This journal is © The Royal Society of Chemistry 2024

Electronic Supplementary Information
Use of a head-to-tail peptide cyclase to prepare hybrid RiPPs

Tung Le,[a] Dongtianyu Zhang,[a] Rachel Martini,[b] Subhanip Biswas,[a] and Wilfred A. van der Donk[a,b,*]

[a]Department of Chemistry and Howard Hughes Medical Institute, University of Illinois at Urbana–Champaign, 600 S. Mathews Ave, Urbana, IL 61801, USA
[b]Department of Biochemistry, University of Illinois at Urbana–Champaign, 600 S. Mathews Ave, Urbana, IL 61801, USA
*Correspondence: vddonk@illinois.edu (W.A. van der Donk); phone: (217) 244-5360, fax: (217) 244-8533.

Table of contents

Materials and methods 4
Liquid chromatography – Mass spectrometry 4
Materials for cloning 4
Plasmid constructs 5
Molecular cloning 5
Strains and culturing 6
General peptide expression and purification 6
Expression and purification of OaAEP1 (C247A) 7
Purification of native subtilosin A 8
Design strategy for generating subtilosin A using OaAEP1 8
Expression and purification of SboA(NGL) and its variants 9
Protease digest of SboA(NGL) and its variants 9
OaAEP1 cyclization of SboA_NGL and its variants 9
2-PCA reaction with native subtilosin A and OaAEP-1-cyclized SboA(NGL)(-N1) 9
HPLC purification of SboA(NGL) and its variants 10
LC-MS/MS analysis of SboA variants 10
Expression and purification of ProcA2.8(15RGD)_NGL and ProcA2.8(16RGD)_NGL 11
LahT digest and HPLC purification of ProcA2.8(15RGD)_NGL and ProcA2.8(16RGD)_NGL 11
OaAEP1 cyclization of ProcA2.8(15RGD)_NGL and ProcA2.8(16RGD)_NGL 11
HPLC purification of ProcA2.8(15RGD)_NGL and ProcA2.8(16RGD)_NGL 11
Fluorescence polarization competition of ProcA2.8(15RGD)_NGL and ProcA2.8(16RGD)_NGL binding to integrin 12
Expression and purification of LctA_NGL 12
Protease digest of LctA(NGL) 13
OaAEP1 cyclization of LctA_NGL core 13
HPLC purification of LctA(NGL) and its variants 13
OaAEP1 cyclization of LctA_NGL core and trypsin digestion 13
Minimum Inhibitory Concentration measurement of lacticin 481 and cyclic variant 13
Expression and purification of $SunA_{core}$_NGL 14
Alkylation of $SunA_{core}$_NGL 15

OaAEP1 cyclization of $SunA_{core}$_NGL 15
LC-MS/MS analysis of sublancin variants 15
Determination of the MIC for sublancin and its variants 15
Supplementary Figures and Tables 16
Protein sequences 29
References 31

Materials and methods

Liquid chromatography – Mass spectrometry

Prep-scale RP-HPLC was performed on a Shimadzu HPLC Nexera system using (H_2O + 0.1%TFA) and (MeCN + 0.1%TFA) as solvent system.

MALDI-TOF MS was carried out on a Bruker Daltonics UltrafleXtreme MALDI TOF/TOF instrument or Bruker Autoflex MALDI TOF. Super DHB (Sigma) was used as MALDI matrix.

LC-ESI-Q/TOF MS (ESI, electrospray ionization; Q: quadrupole) and MS/MS analyses were conducted using an Agilent Infinity1260 LC coupled to an Agilent 6545 LC/QTOF instrument, using (H_2O + 0.1%FA) and (MeCN + 0.1%FA) as solvent system. The general qTOF conditions were:

Polarity	Positive
Data storage	Centroid
Acquisition range (m/z)	100-1700
Gas temp (ºC)	320
Gas Flow (l/min)	13
Nebulizer (psig)	35
Sheath gas temp	275
Sheath gas flow	12
Vcap	3500
Nozzle Voltage	1000
Fragmentor (V)	125
Skimmer	65
Octopole RFPeak	750
Scan rate (spectra/s)	10

MS/MS spectra were matched using Interactive peptide spectral annotator.[1]

Materials for cloning

Oligonucleotides were obtained from Integrated DNA Technologies. Cloning was performed using restriction enzymes, Q5 polymerase (for PCR), NEBridge® Golden Gate Assembly BsaI-HF (for Golden Gate assembly), and NEB Hifi DNA Assembly (for Gibson isothermal assembly) purchased from New England Biolabs (NEB). All polymerase chain reactions were carried out on a C1000 thermal cycler (Bio-Rad). DNA sequencing was performed by ACGT, Inc. Antibiotics for culturing cells were purchased from GoldBio. Media components were purchased from Bacto™ (Thermo Fisher).

Plasmid constructs

Plasmid	Source
pRSF : His-ProcA2.8(15RGD) : ProcM	Ref. [2]
pRSF : His-ProcA2.8(16RGD) : ProcM	Ref. [2]
pRSF : His-ProcA2.8(15RGD)_NGL : ProcM	This study
pRSF : His-ProcA2.8(16RGD)_NGL : ProcM	This study
pRSF : His-lctA : lctM	Ref. [3]
pRSF : His-lctA_NGL : lctM	This study
pRSF : His6_SUMO_tev_SunA$_{core}$_NGL: SunS	This study
pETDuet : His-SboA : AlbA	This study
pETDuet : His-SboA_NGL : AlbA	This study
pETDuet : His-SboA(insR2)_NGL : AlbA	This study

Molecular cloning

Cloning of pRSF : His-ProcA2.8(15RGD)_NGL : ProcM and pRSF : His-ProcA2.8(16RGD)_NGL : ProcM

Plasmids pRSF : His-ProcA2.8(15RGD) : ProcM and pRSF : His-ProcA2.8(16RGD) : ProcM were used as templates for PCR using primers:

p28(15RGD)_NGL_R	gcggccgcttaAAGACCATTgcactcatcgccacgatag
p28(15RGD)_NGL_F	gcgatgagtgcaatGGTCTTtaagcggccgcataatgc
p28(16RGD)_NGL_R	gccgcttaCAGACCATTgcaatcaccacgccaatag
p28(16RGD)_NGL_F	gtgattgcAATGGTCTGtaagcggccgcataatgc

The two PCR products were gel-purified and re-assembled using Gibson assembly

Cloning of pRSF : His-lctA_NGL : lctM

Plasmid pRSF : His-lctA : lctM was used as template for PCR using primers:

lctA_NGL_R	gcggccgcttaAAGACCATTagagcagcaagtaaatacaaattgcc
lctA_NGL_F	cttgctgctctAATGGTCTTtaagcggccgcataatgctt

The PCR product was gel-purified and assembled using Gibson assembly.

Cloning of pETDuet : His-SboA : AlbA and variant constructs

All pETDuet backbones used in this study had the BsaI cut site removed for Golden Gate cloning. To achieve this, plasmid pET-Duet was amplified with primers:

pETDuet-xBsaI_R	AACGACGGTCTCGtcGcgcggtatcattgcagca
pETDuet-xBsaI_F	AACGACGGTCTCGgCgacccacgctcaccggct

The PCR product was gel-amplified and re-assembled with Golden Gate cloning to yield pETDuet(xBsaI) plasmid free of BsaI cut site.

pETDuet : His-SboA : AlbA

For preparing the backbone, pETDuet(xBsaI) was amplified with primers:

pETDuet-xBsaI FWD (1)	AGCAAGGGTCTCGgtaattaacctaggctgctgcc
pETDuet-xBsaI REV (1)	AGCAAGGGTCTCGttcatcgaattcggatcctggct
pETDuet-xBsaI FWD (2)	AGCAAGGGTCTCGgataataatgcttaagtcgaacagaaagt
pETDuet-xBsaI REV (2)	AGCAAGGGTCTCGacatatgtatatctccttcttatacttaactaatatact

The two PCR products were gel-purified.
For inserts, *B. subtilis* 168 was cultured in 5 mL of LB media, followed by gDNA extraction with PureLink™ Genomic DNA Mini Kit (ThermoFisher). The gDNA was used as template for PCR reactions with primers:

SboA BGC FWD (1)	AGCAAGGGTCTCGtgaaaaaagctgtcattgtgga
SboA BGC REV (1)	AGCAAGGGTCTCGtatccccatagaccgaatagac
SboA BGC FWD (2)	AGCAAGGGTCTCGatgtttccatttattaatgaaagtgtaaga
SboA BGC REV (2)	AGCAAGGGTCTCGttactaaataagctggaccacgtc

to amplify *sboA* and *albA* genes. The two PCR products were gel-purified
All four fragments from above were assembled with Golden Gate assembly to yield pETDuet : His-SboA : AlbA

Cloning variants of pETDuet : His-SboA : AlbA

pETDuet : His-SboA : AlbA was amplified with the following primer pairs:

SboA(NGL)_F	ATTGGCGGTCTCCGCCTTTAAtaatgcttaagtcgaacagaaagt
SboA(NGL)_R	ATTGGCGGTCTCCAGGCCATTtccccatagaccgaatagac

The PCR product was gel-purified and re-assembled using Golden Gate assembly to yield pETDuet : His-SboA_NGL : AlbA. This plasmid was then amplified with primers:

SboA(insR2)_R	CTACGTGGTCTCCcggttttctacaatgacagcttttttcatc
SboA(insR2)_F	CTACGTGGTCTCCaccgtaaaggttgtgcaacatgct

The two PCR products were gel-purified and re-assembled with Golden Gate assembly to yield corresponding variant constructs.

Strains and culturing

Unless stated otherwise, *E. coli* NEB Turbo and *E. coli* BL21(DE3)-T1^R were used as hosts for cloning and protein expression, respectively. Cells were cultured using LB or TB media supplemented with appropriate antibiotics at the following concentration: ampicillin/carbenicillin (100 µg/mL), kanamycin (50 µg/mL), chloramphenicol (25 µg/mL).

General peptide expression and purification

Start buffer	100 mM NaH_2PO_4, 10 mM Tris, 6 M guanidine, 10 mM imidazole, 500 mM NaCl, pH 8.0
Wash buffer	100 mM NaH_2PO_4, 10 mM Tris, 6 M guanidine, 50 mM imidazole, 500 mM NaCl pH 8.0
Elution buffer	20 mM HEPES, 500 mM imidazole, 300 mM NaCl, pH 8.0

Plasmid containing His-tagged peptide was used to transform *E. coli* BL21 T^{1}R. Several colonies from overnight plates were used to inoculate 5-10 mL cultures of media (+antibiotics), cultured at 37 ºC for 5-6 h until visible growth, then sub-cultured to 500 mL of media (+antibiotics) and cultured at 37 ºC, at 220 rpm. When the OD_{600} reached ~1-2, expression in the culture was induced with 0.25 mM IPTG, and the cells were cultured at 18 ºC, 220 rpm for further 16-20 h. Cells were harvested by centrifugation at 5,000xG, 15 min, resuspended in Start buffer at 5 mL per gram cell pellets and stored at -70 ºC until use.

Purification was performed at room temp. Cells were lysed by sonication on ice (Vibra Cells, 50% amplitude, 10 s ON – 10 s OFF, total 4 min ON time. Lysate was clarified by centrifugation at 12,000xG for 30 min, then carefully decanted into Ni resin (His6 Takara Superflow resin, 2 mL resin / L culture) pre-equilibrated in Start buffer. The resuspension was slowly rotated for 30-45 min. Resin was collected by centrifugation at 1,000xg for 5 min, then applied to empty gravity column. Resin was washed with 20-30 column volumes (CV) of Wash buffer until $A_{280} < 0.05$. Peptide was eluted by ~4 CV of Elution Buffer.

Expression and purification of OaAEP1 (C247A)

His-tagged Ulp-1 protease (also called SUMO protease) was a gift from the Nair lab at UIUC. pBHRSF184 was a gift from Hideo Iwai (Addgene plasmid # 89482 ; http://n2t.net/addgene:89482 ; RRID:Addgene_89482).[4] This plasmid was used as template for QuikChange mutagenesis to mutate Cys247 residue of OaAEP1 to Ala, which was shown to improve the kinetic efficiency of this enzyme.[5] The resulting plasmid was called pBHRSF184 : His6_SUMO_OAAEP (C247A). This plasmid was used to transform Shuffle T7 *E. coli* (NEB). Single colony was cultured overnight, followed by subculturing into 2 L of LB(+kan) (37 °C, 200 rpm). When OD_{600} reached 0.6-0.8, the culture was cooled in an ice-water bath for 5-10 min and induced with 0.25 mM IPTG, then returned to the shaker (18 °C, 200 rpm) for 18-20 h, resulting in ~4-5 g cell pellet/L culture.

Buffer A	20 mM HEPES, 500 mM NaCl, 10% glycerol, pH 8.0
Buffer B	20 mM HEPES, 300 mM NaCl, 300 mM imidazole, 10% glycerol, pH 8.0
Ulp-1	20 mM HEPES, 150 mM NaCl, 0.5 mM DTT, 10% glycerol, pH 8.0

Cell pellet was harvested at 5000xg, 10 min and resuspended (5 mL/g pellet) in Buffer A + 10 mM imidazole. To ensure lysis, cells were passed three times through an Avestin Homogenizer C3 (10,000-15,000 psi) and clarified by centrifugation at 12,000xg for 30 min. The supernatant was decanted into Ni resin (2 mL resin / L culture), and slowly rotated in a cold room for 30-45 min.The resin was collected by centrifugation at 1,000xG for 5 min, then loaded to an empty gravity column (Bio-Rad). The column was washed with 4-5 CV of ATP wash buffer (Buffer A + 5 mM $MgCl_2$, 5 mM ATP, 50 mM imidazole), followed by Wash buffer (Buffer A + 50 mM imidazole) until A280 was < 0.05. The ATP wash would release any bound chaperone protein (DnaK).[6] Protein was eluted with 4 CV of Buffer B, concentrated using an Amicon 50K MWCO and the buffer was exchanged to Ulp-1 buffer using a PD-10 Column (Cytiva). The yield of protein was 5-6 mg/L culture. To the protein solution (~50 µM), His-tagged Ulp-1 protease was added to 0.2 µM final concentration and incubated at RT for 2 h to cleave off SUMO tag. After cleavage was completed, the reaction was concentrated using an Amicon 30K MWCO and the protein was further purified with Size-Exclusion Chromatography:

Column	Superdex 75 Increase 10/300GL
SEC buffer	20 mM HEPES, 150 mM NaCl, pH 8.0
Flow rate	0.4 mL/min

Zymo-OaAEP1 eluted after 10 mL.

To activate autolysis activity of OaAEP1, TCEP (0.5 mM final) and EDTA (1 mM final) was added to Zymo-OaAEP1 protein solution. Next, aliquots of 1 M aqueous NaOAc pH 3.6 was added to acidify the solution such that pH < 4.5 (this usually requires 1:10 to 1:20 volume), followed by

incubation at RT overnight. After this period, there would be white flocculent which contained some precipitated proteins. The pH was brought to ~ 6-7 by addition of aliquots of 1 M HEPES pH 7.0 followed by centrifugation to remove the precipitate.

Finally, the supernatant, which contained core OaAEP1 and the cap domain, was concentrated using Amicon 10K MWCO and stored at -70 ºC.

Purification of native subtilosin A

Purification of native subtilosin A was performed following a published procedure with slight modification.[7] For 1-L culture scale, 25 g of Amberlite XAD16 resin (Fisher) was soaked in 10 vol of water for 30 min, followed by 10 vol of MeOH for 30 min to swell the resin. Then the resin was soaked in 10 vol of water and the water drained before use.

B. subtilis 168 was cultured overnight in LB media at 37 ºC, then sub-cultured (1:100) into 1 L of LB media at 37 °C and 220 rpm until an OD_{600} of 0.8 was reached. Afterwards the shaking was reduced to 100 rpm and the cells were cultured for additional 24 h. Following centrifugation at 5000xg for 10 min, the culture supernatant was collected and acidified by addition of TFA to 0.5% final concentration. The supernatant was clarified again at 5000xg for 10 min, then decanted into 25 g of XAD16 resin prepared as described above. The suspension was shaken at 100 rpm and room temperature overnight. The next day, the resin was collected by applying vacuum through a 600-mL glass frit, then washed twice with 200 mL of DI water. The compound was eluted by addition of 100 mL of MeOH with intermittent swirling for 15-20 min. The elution step was repeated once. The eluent fractions were combined and evaporated with a rotavap until a brown residue remained. The residue was dissolved with 100 mL of n-butanol:water (1:1 vol). The upper organic phase was collected, evaporated with a rotovap to almost dryness (cloudy yellow suspension). The suspension was dissolved with 30-40 mL of methanol:water (1:1 vol), filtered and further purified with a RP-HPLC system.

Solvent	A: Water + 0.1% TFA<br>B: MeCN + 0.1% TFA
Column	MacNagel® 5 µm C18 100 Å, LC Column 250 x 10 mm
Gradient	4 mL/min<br>0-5 min: 10-40% B<br>5-25 min: 40-60% B<br>25-30 min: 60-80% B

The collected fractions were checked with MALDI-TOF MS. Under this condition, subtilosin A elutes around 27 min. The final yield was ~ 0.6 mg per L culture.

Design strategy for generating subtilosin A using OaAEP1

N-to-C cyclized natural product peptides have a specific site of enzymatic cyclization, but after cyclization that site becomes traceless. Hence, as has been pointed out previously,[8] alternative sites of cyclization are possible by designing the cyclization substrate such that upon cyclization an identical structure is formed (circular permutation of the cyclization site). In our case, subtilosin is

naturally cyclized by amide bond formation between the amino group of Asn1 and the carboxylate of Gly35 (Fig. 2, main text). Since the OaAEP enzyme recognizes the NGL sequence and cleaves after Asn, we reasoned that the subtilosin ring could also be made by cyclization of the amino group of Lys2 and the carboxylate of an Asn placed at position 36 with the removal of Asn1 (Fig. 2).

Expression and purification of SboA(NGL) and its variants
Expression was done using LB media (+amp/cam) supplemented with Trace Metal mix (Teknova T1001). *E. coli* BL21-DE3(T1^R) was co-transformed with pETDuet : His-SboA_NGL : AlbA and pACYC-sufABCDSE, which contained genes for assembly of Fe-S clusters. Overnight culture from single colony was sub-cultured (1:200) into LB media, and cultured at 37 °C, 220 rpm for 2-3 h until OD_{600} ~ 0.5-0.7. The culture was incubated on ice for 5-10 min, induced with 0.25 mM IPTG and cultured at 18 °C, 80 rpm overnight (18-20 h). Cell pellet was harvested and purified according to the protocol in *General peptide expression and purification*. Eluted peptide was desalted with a C8 SPE column (ThermoFisher HyperSep, 500 mg resin bed) and lyophilized to dryness. Final yield of full-length peptide was ~3 mg/L culture.

Protease digest of SboA(NGL) and its variants

- Lys-N digestion: Full-length SboA peptide was dissolved in water to 100 µM. HEPES 1 M pH 7.0 was added to 100 mM final concentration, followed by Lys-N to 1 µM final concentration. The reaction was incubated at 37 °C for 3 h.
- Arg-C digestion: Full-length peptide was dissolved in water to 100 µM and sonicated for 5 min to facilitate solubilization. Then 10X buffer (500 mM Tris pH 7.6, 10 mM $CaCl_2$) was added to 1X, followed by 1 M DTT to 10 mM final concentration. Arg-C was added to 1-2 µM. The reaction was incubated at 37 °C for 2 h.
- Lys-C digestion of native subtilosin A and cyclized products from OaAEP-1 cyclization of SboA(NGL)(-N1): Native subtilosin A and the two cyclized products obtained from OaAEP-1 cyclization of SboA(NGL)(-N1) (shown in Fig. S1a) were dissolved in 50 mM HEPES, pH 7.0 to 100 µM final concentration. Lys-C was then added to 2 µM final concentration and the reaction was incubated at 37 °C for 2 h. Presence of hydrolyzed product for each peptide was checked with MALDI-TOF MS.

OaAEP1 cyclization of SboA_NGL and its variants
Core peptide was dissolved in 20 mM sodium phosphate, pH 6.0 to 50-100 µM final concentration. OaAEP1 was added to 1 µM and the reaction was incubated at 37 °C for 1 h.

2-PCA reaction with native subtilosin A and OaAEP-1-cyclized SboA(NGL)(-N1)
2-Pyridinecarboxaldehyde (2-PCA) is known to react exclusively with an exposed N-terminus of a peptide, and not with a lysine side chain, in part because the initial imine adduct can cyclize with the amide of the residue at position 2 of the peptide chain.[9] Native subtilosin A and the cyclized isomers obtained from OaAEP-1 cyclization of SboA(NGL)(-N1) on HPLC (shown in Fig. S1a) were dissolved in 20 mM sodium phosphate (pH 7.5) to 20 µM final concentration, followed by 2-pyridinecarboxaldehyde (2-PCA) to 2 mM and the reaction was incubated at 37 °C for 90 min. The reaction was acidified by the addition of 10% formic acid to 1% final concentration, and analyzed by MALDI-TOF MS. The reaction was stopped prior to completion because

transamination products of the starting material, containing an N-terminal aldehyde (M-1 Da), were observed. Such products were not observed for a standard synthetic peptide and have not been reported in other studies using 2-PCA. Apparently, the cyclization step with the amide of the second residue that makes 2-PCA selective for the N-terminal amine is slow in the context of subtilosin compared to linear peptides, which is likely caused by the constrained structure of the mature product (Figure S1e). When the cyclization step is slow, isomerization of the initially formed imine between 2-PCA and the starting peptide and hydrolysis leads to N-terminal aldehyde formation.

Solvent	A: 0.1% formic acid<br>B: MeCN + 0.1% formic acid
Column	Kinetex® 2.6 µm C8 100 Å, LC Column 150 x 2.1 mm
Gradient	0.4 mL/min, 45 ºC, 10 µL injection<br>0-3 min: 5% B<br>3-18 min: 5-95% B

The ion chromatogram from 10.8 to 13.6 min, which spans the elution time of all relevant species, was extracted and deconvoluted.

HPLC purification of SboA(NGL) and its variants

When enzymatic reaction was completed, reaction was diluted (1:1 volume) with MeOH, centrifuged to remove insoluble material and injected onto a RP-HPLC system using following conditions:

Solvent	A: Water + 0.1% TFA<br>B: MeCN + 0.1% TFA
Column	MacNagel® 5 µm C18 100 Å, LC Column 250 x 10 mm
Gradient	4 mL/min<br>0-5 min: 2-30% B<br>5-25 min: 30-80% B

Core peptides generally eluted between 15-20 min.

LC-MS/MS analysis of SboA variants

The sample was acidified by addition of 10% TFA to 1% final concentration, centrifuged to remove insoluble debris and injected to a LC-MS system. General qTOF condition was used.

Solvent	A: 0.1% formic acid<br>B: MeCN + 0.1% formic acid
Column	Kinetex® 2.6 µm C8 100 Å, LC Column 150 x 2.1 mm

Gradient	0.4 mL/min, 45 ºC, 10 µL injection<br>0-2 min: 5-50% B<br>2-7 min: 50-95% B

For MS/MS analysis, the qTOF was run in targeted mode, fragmenting $[M+3H]^{3+}$ precursor ions using collision voltage of 20-25 V.

Expression and purification of ProcA2.8(15RGD)_NGL and ProcA2.8(16RGD)_NGL
Expression was done using TB media (+kan). BL21-DE3($T1^R$) was transformed with corresponding co-expression plasmids, cultured, harvested and purified according to the protocol in *General peptide expression and purification*. The eluant fraction was kept and proceeded to LahT digest.

LahT digest and HPLC purification of ProcA2.8(15RGD)_NGL and ProcA2.8(16RGD)_NGL
The buffer of the eluant fraction from Ni purification was exchanged to LahT150 buffer (20 mM HEPES pH 8.0, 0.5 mM TCEP) using an Amicon 3K MWCO, making sure the imidazole concentration was < 50 mM. LahT150[10] was added to 5 µM final concentration and the reaction was incubated at RT overnight. To quench the reaction, 10% TFA stock was added to 0.5% final concentration. The reaction was filtered and injected onto a RP-HPLC system using the following condition:

Solvent	A: Water + 0.1% TFA<br>B: MeCN + 0.1% TFA
Column	Phenomenex Luna 5 µm C5 100 Å, LC Column 250 x 10 mm
Gradient	4 mL/min<br>0-40 min: 2-80% B

The core peptide generally elutes around 20-25 min.

OaAEP1 cyclization of ProcA2.8(15RGD)_NGL and ProcA2.8(16RGD)_NGL
The core peptide was dissolved in 20 mM aqueous sodium phosphate, pH 6.0 to 50-100 µM final concentration. OaAEP1 was added to 1 µM and the reaction was incubated at 37 ºC for 1 h.

HPLC purification of ProcA2.8(15RGD)_NGL and ProcA2.8(16RGD)_NGL
When the enzymatic reaction was completed, the reaction was diluted (1:1 volume) with MeOH, centrifuged to remove insoluble material and injected onto a RP-HPLC system using the following conditions:

Solvent	A: Water + 0.1% TFA<br>B: MeCN + 0.1% TFA
Column	MacNagel® 5 µm C18 100 Å, LC Column 250 x 10 mm
Gradient	4 mL/min<br>0-40 min: 2-80% B

Fluorescence polarization competition of ProcA2.8(15RGD)_NGL and ProcA2.8(16RGD)_NGL binding to integrin

Binding affinity of ProcA2.8 variants for αβV3 integrin was determined via a fluorescence polarization competition assay as previously reported.[2] In brief, peptide was titrated against fixed concentration of αβV3 integrin (R&D Systems) and fluorescein-c(RGDyK) (Anaspec). At equilibirum, fluorescence polarization was measured and the K_i was then determined from the IC_{50} value and K_D value of probe and target protein. The experimental details are as followed.

2X Integrin Binding Buffer (2X IBB)	Concentration	Unit
Tris, pH 8.0	50	mM
NaCl	300	mM
$CaCl_2$	4	mM
$MgCl_2$	2	mM
$MnCl_2$	2	mM
BSA	0.2	%

2X RGD-integrin sample (10 nM fluorescein-c(RGDyK) – 30 nM integrin) was prepared in 2X Integrin Binding Buffer. In a black 96-well half-area plate (Corning 3686), 20 µL of 200 µM peptide stock in PBS was added to the first well, followed by 10 2-fold dilutions into PBS buffer at subsequent wells. 20 µL of 2X RGD-integrin stock was added to each well. The plate was covered from light and shaken at RT. Fluorescence polarization was measured (Biotek Synergy H4 hybrid reader) every 15 min using the following filter (Excitation : Emission – 485 nm/20 nm : 518 nm/20 nm).

The obtained data was converted to anisotropy value and plotted against peptide concentration and fitted to a dose response function using Origin Pro software:

$$y = A1 + \frac{(A2 - A1)}{1 + (\frac{IC_{50}}{x})^p}$$

Where: y = anisotropy value, A1 = minimum anisotropy, A2 = maximum anisotropy, p = Hill's coefficient (fixed at -1), x = competitor concentration. The obtained IC_{50} was used to calculate inhibition constant K_i using the Cheng-Prussoff equation:

$$K_i = \frac{IC_{50}}{1 + \frac{S}{K_D}}$$

Where: S is probe concentration, and K_D is the binding constant of probe (13 nM).

Expression and purification of LctA_NGL

Expression was done using TB media (+kan). *E. coli* BL21-DE3(T1^R) was transformed with pRSF : His-lctA_NGL : lctM. Cells were cultured, harvested and purified according to the protocol in

General peptide expression and purification. Eluted peptide was desalted with a C8 SPE column (ThermoFisher HyperSep, 500 mg resin bed) and lyophilized to dryness.

Protease digest of LctA(NGL)

Trypsin digestion: Full-length lctA_NGL peptide was dissolved in 50 mM NH_4HCO_3 to 100 µM. Trypsin was added to 1-2 µM. The reaction was incubated at 37 ºC for 4 h.

OaAEP1 cyclization of LctA_NGL core

Core peptide was dissolved in 20 mM sodium phosphate, pH 6.0 to 50-100 µM final concentration. OaAEP1 was added to 1 µM and the reaction was incubated at 37 ºC for 1 h.

HPLC purification of LctA(NGL) and its variants

When the enzymatic reaction was completed, the reaction was diluted (1:1 volume) with MeOH, centrifuged to remove insoluble material and injected onto a RP-HPLC system using the following conditions:

Solvent	A: Water + 0.1% TFA<br>B: MeCN + 0.1% TFA
Column	MacNagel® 5 µm C18 100 Å, LC Column 250 x 10 mm
Gradient	4 mL/min<br>0-40 min: 2-80% B

OaAEP1 cyclization of LctA_NGL core and trypsin digestion

Core peptide was dissolved in 20 mM sodium phosphate, pH 6.0 to 40 µM final concentration. OaAEP1 was added to 5 µM and the reaction was incubated at 37 ºC for 24 h. 10-µL aliquot was collected and mixed with 1 µL of 0.5 M ammonium bicarbonate, followed by trypsin to 1-2 µM. The reaction was continued at 37 ºC for 1 h, quenched by mixing 1:1 with 1% TFA and ziptiped for MALDI analysis.

Minimum Inhibitory Concentration measurement of lacticin 481 and cyclic variant

The MIC experiment was performed with *L. lactis* subsp. cremoris as the indicator strain,[11] and GM17 as culture media. Stock peptide was prepared in water at 20 µM. In a clear Nuclon 96-well plate, 50 µL of peptide solution was added to the first well, followed by nine 2-fold serial dilutions into water in the subsequent wells. Overnight bacteria culture was diluted to $OD_{600} = 0.1$ using 2X GM17 media. Then, 50 µL of this culture was added to each peptide sample well, resulting in $OD_{600} = 0.05$ and 1X GM17 media final concentration. A well with culture and media was used as positive growth control, and another well with water and media was used as negative growth control. The plate was covered with a clear lid and incubated at 30 ºC using a BioTek Hybrid plate reader (H4) for 24 h. OD_{600} was taken every hour after briefly shaking for 30 s. The OD_{600} values at 18-h timepoint was plotted as function of peptide concentration and fitted using the Dose-Response curve:

$$y = A1 + \frac{(A2 - A1)}{1 + (\frac{IC_{50}}{x})}$$

Where: y = OD value, A1 = minimum OD value, A2 = maximum OD value, x = peptide concentration.

Expression and purification of $SunA_{core}$_NGL

The glycosylated sublancin variant was obtained via co-expression with the glycosyl transferase SunS. In this construct, the SunA core peptide was fused to an N-terminal His-SUMO tag with a TEV cleavage site. As reported previously, the glycosyl transferase SunS still recognizes SunA core peptide without the leader sequence and catalyzes the glycosylation reaction.[12]

Plasmid pRSF : His6_SUMO_tev_$SunA_{core}$_NGL: SunS was used to transform *E. coli* K12 SHuffle cells. The cells were plated on LB agar plates with 50 mg/L kanamycin, and grown overnight at 37 °C. A single colony was picked from the plate to inoculate 5 mL of LB media with 50 mg/L kanamycin and 100 mg/L spectinomycin. The small culture was grown at 37 °C overnight, and used to inoculate 1 L of terrific broth containing 50 mg/L kanamycin. The culture was grown at 30 °C with shaking at 195 RPM, till the OD600 reached 0.8. The culture was cooled on ice for 20 min, then induced with IPTG to a final concentration of 500 µM. The cells were cultured at 18 °C for 16 h, then harvested by centrifugation at 8,000 xg for 10 min, and frozen at -70 °C.
Purification was performed at room temp. Cells were resuspended in buffer B1. Cells were lysed by sonication on ice (Vibra Cells, 50% amplitude, 2 s ON – 5 s OFF, total 4 min ON time). Lysate was clarified by centrifugation at 45,000 xg for 30 min. Supernatant was filtered with a 0.45 micron filter and loaded onto a Ni resin (His6 Takara Superflow resin, 3 mL resin /L culture) column equilibrated in B1 buffer. Resin was washed with 3 CV of buffer B2 then eluted with 4 CV of elution buffer (EB).

Buffer B1	20 mM sodium phosphate, 500 mM sodium chloride, 0.5 mM imidazole, 10% glycerol, pH 7.5
Buffer B2	20 mM sodium phosphate, 300 mM sodium chloride, 15 mM imidazole, pH 7.5
EB	20 mM sodium phosphate, 150 mM sodium chloride, 500 mM imidazole, pH 7.5

The buffer of the eluted protein was exchanged to TEV buffer (50 mM Tris pH 8.0, 0.5 mM EDTA, 3 mM glutathione, 0.3 mM oxidized glutathione) using an Amicon 3K MWCO, making sure the imidazole concentration was < 50 mM. TEV protease was added to 2-3 µM and the reaction was incubated at RT for 2 h. The reaction was quenched by adding TFA to 1% final concentration, filtered, and injected onto a RP-HPLC system for further purification:

Solvent	A: Water + 0.1% TFA<br>B: MeCN + 0.1% TFA
Column	Phenomenex 10 µm C5 100 Å, LC column 250 x 10 mm
Gradient	4 mL/min<br>0-5 min: 2-30% B<br>5-35 min: 30-60% B

The peptide eluted around 15 min.

Alkylation of $SunA_{core}$_NGL

The purified peptide was resuspended in alkylation buffer (50 mM HEPES pH 8.5, 5 mM TCEP) at 100 µM. After 30 min at RT, aliquots of a stock solution of 100 mM IAA were added to obtain 20 mM final concentration. The reaction was incubated at RT in the dark for 30 min. The reaction was quenched by adding TFA to 1% final concentration before further analysis by MALDI-TOF MS and ESI-MS.

OaAEP1 cyclization of $SunA_{core}$_NGL

The core peptide was dissolved in 20 mM sodium phosphate, pH 6.0 to 50-100 µM final concentration. OaAEP1 was added to 5 µM and the reaction was incubated at RT overnight. The next day, the reaction was quenched by adding TFA to 0.5% final concentration, cleaned up by C18 SPE column (Agilent Bond Elut) and eluted into 60% MeCN. The eluant was diluted 2-fold with water and injected onto an analytical RP-HPLC system for further purification:

Solvent	A: Water + 0.1% TFA<br>B: MeCN + 0.1% TFA
Column	MacNagel 5 µm C18 100 Å, LC Column 250 x 10 mm
Gradient	4 mL/min<br>0-5 min: 2-30% B<br>5-35 min: 30-80% B

The peptide eluted around 17 min.

LC-MS/MS analysis of sublancin variants

The sample was acidified by addition of 10% TFA to 1% final concentration, centrifuged to remove insoluble debris and injected onto an LC-MS system. The general qTOF condition was used.

Solvent	A: 0.1% formic acid<br>B: MeCN + 0.1% formic acid
Column	Kinetex® 2.6 µm C8 100 Å, LC Column 150 x 2.1 mm
Gradient	0.4 mL/min, 45 ºC, 10 µL injection<br>0-2 min: 5-30% B<br>2-7 min: 30-80% B

For MS/MS analysis, the qTOF MS experiment was run in targeted mode, fragmenting $[M+4H]^{3+}$ precursor ions using a collision voltage of 30-40 V.

Determination of the MIC for sublancin and its variants

MIC determination for sublancin and the cyclized variant was adapted from a published protocol.[12] In a clear Nuclon 96-well plate, 50 µL of peptide solution was added to the first well, followed by nine 2-fold serial dilutions into water in the subsequent wells. Overnight *B. subtilis 168 ΔSPβ* culture was diluted to OD_{600} = 0.1 using LB media. Then, 50 µL of this culture was added to each peptide sample well, resulting in final OD_{600} = 0.05. A well with culture and media was used as

positive growth control, and another well with water and media was used as negative growth control. The plate was shaken with opened lid at 30 ºC using a BioTek Hybrid plate reader (H4) for 6 h and the OD_{600} was taken every hour. Then, the plate was covered with a lid and shaken at 30 ºC for additional 12 h. The OD_{600} values at 6-h timepoint was plotted as function of peptide concentration and fitted using the Dose-Response curve:

$$y = A1 + \frac{(A2 - A1)}{1 + (\frac{IC_{50}}{x})}$$

Where: y = OD value, A1 = minimum OD value, A2 = maximum OD value, x = peptide concentration. The MIC was determined as the peptide concentration that resulted in 90 % inhibition according to the OD value. The MIC for native sublancin was 1.9 ± 1.0 µM, and the MIC for cyclized sublancin was 2.4 ± 1.0 µM.

Supplementary Figures and Tables

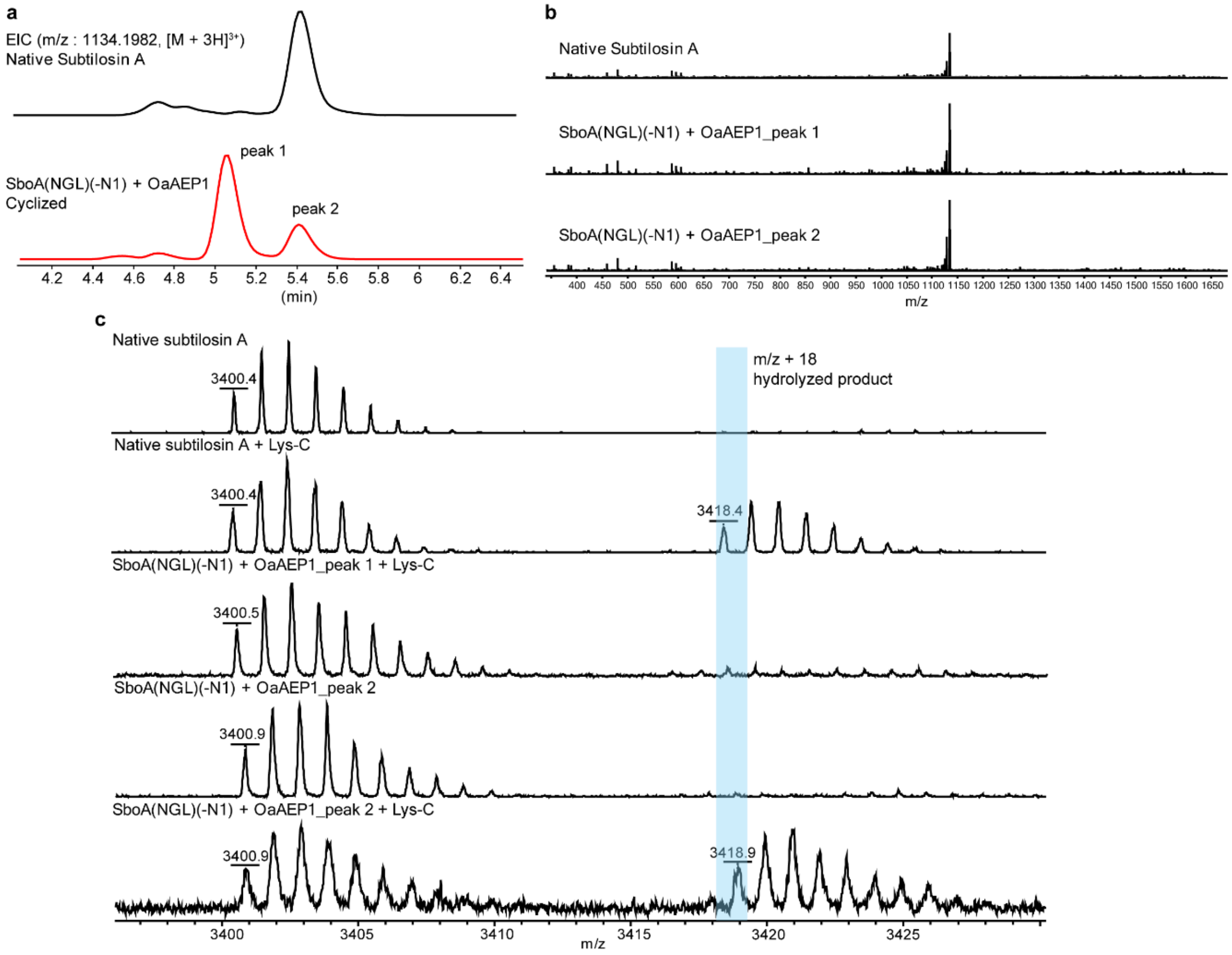

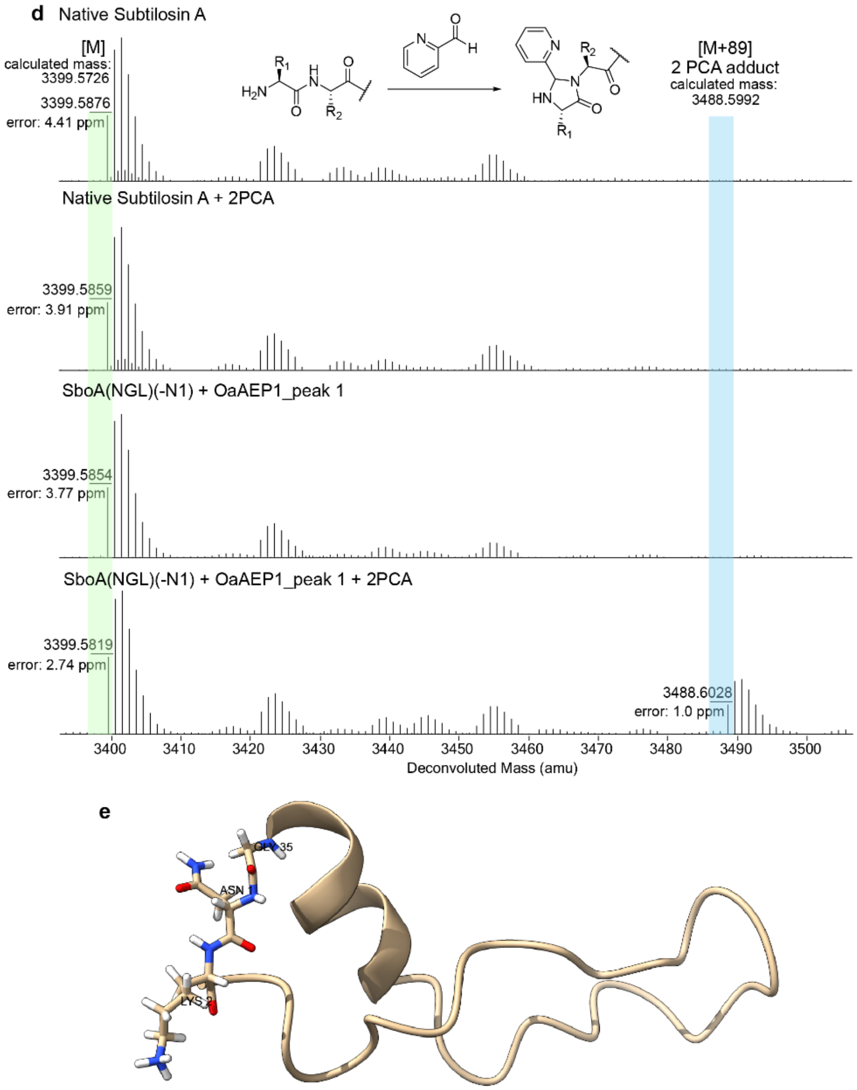

Figure S1. (a) Extracted ion chromatograms of native subtilosin A standard and the products of the OaAEP1 reaction with SboA(NGL)(-N1). (b) MS-MS analysis of native subtilosin A and the two products produced by OaAEP1. Neither product nor the authentic standard resulted in any fragment ions consistent with head-to-tail cyclization. As shown in Fig. S2, the linear analog readily fragments. (c) MALDI-TOF MS analysis of Lys-C digestions of native subtilosin A standard and the products of the OaAEP1 reaction with SboA(NGL)(-N1). Only native subtilosin A and the OaAEP1 peak 2 product were hydrolyzed (M+18), consistent with cleavage of an amide bond inside the macrocycle. Peak 1 peptide was not hydrolyzed by endoproteinase LysC, consistent with the side chain of Lys1 being involved in amide bond formation by OaAEP1. (d) HR-ESI analysis of 2-PCA reaction with native subtilosin A and cyclization isomers from OaAEP-1 cyclization of SboA(NGL)(-N1). The presence of a 2-PCA adduct with peak 1 but not subtilosin A standard indicates a free N-terminal α-amine group in the former, consistent with an iso-peptide cyclized product. (e) NMR structure of subtilosin (PDB 1pxq)[13] where Asn1, Lys2 (N-terminal residue in SboA(NGL)(-N1)), and Gly35 (last residue) are shown in stick format.

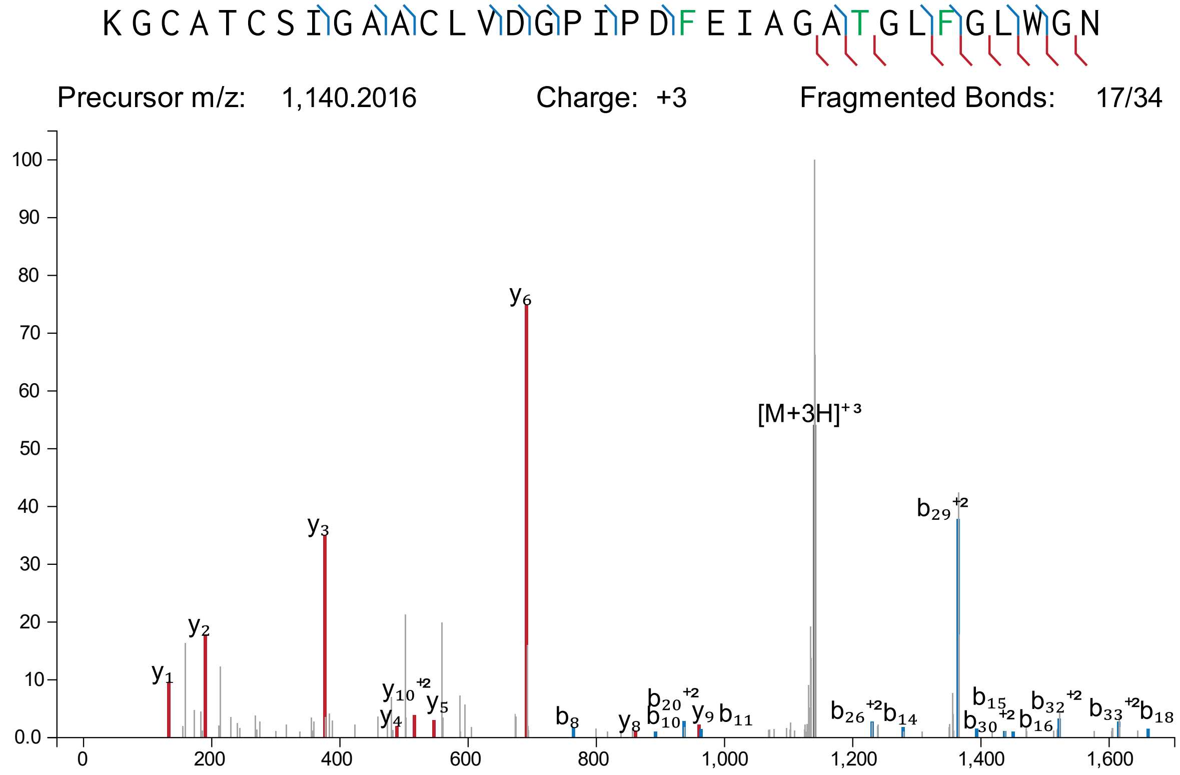

Figure S2. MS/MS analysis of OaAEP1-hydrolyzed product of SboA(NGL)(-N1). Residues in green were matched as dehydrated amino acids, which result from collision-induced breakage and tautomerization of the sactionine linkage.[14] Assignments of b/y ions are shown in Table S1.

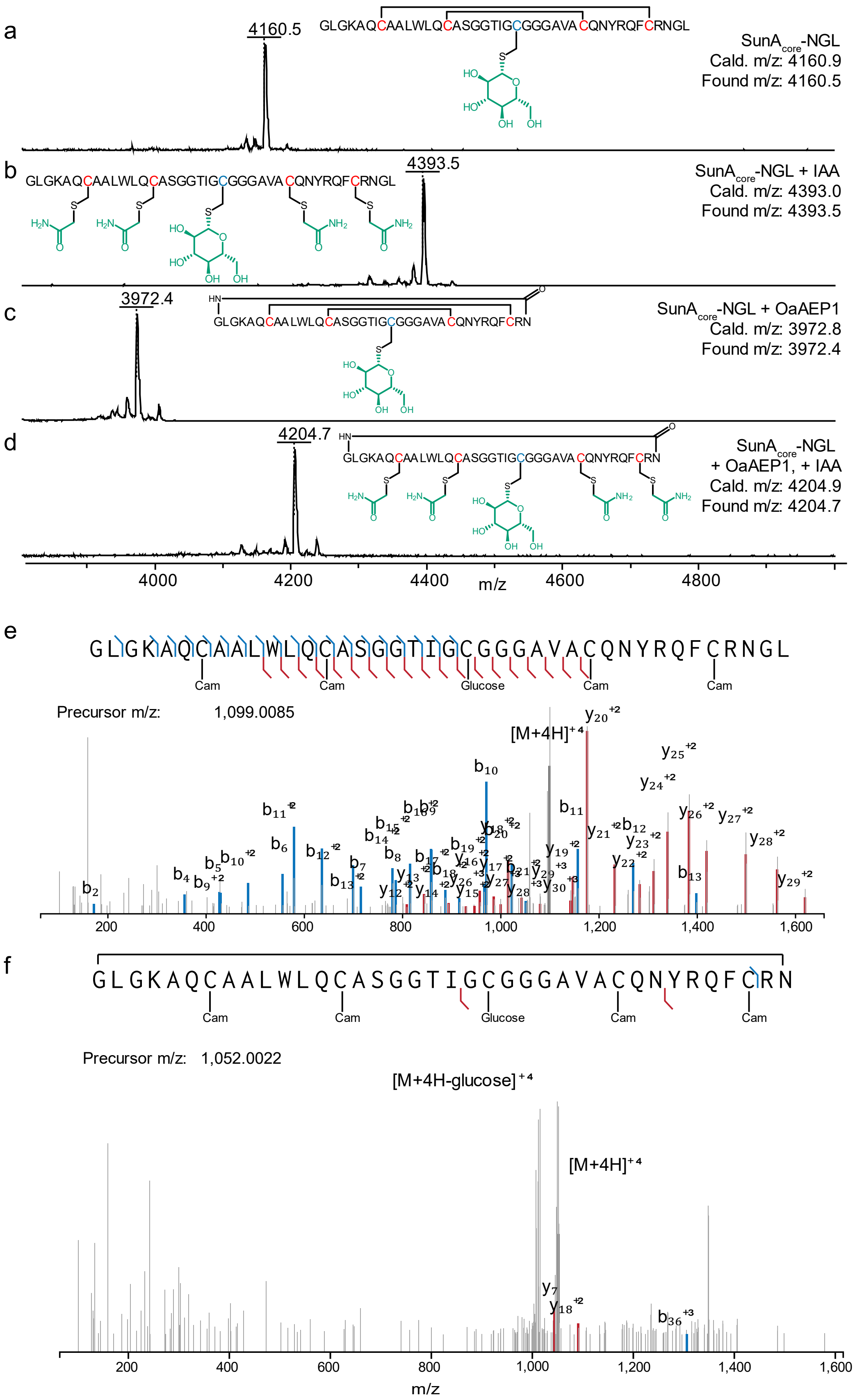

a
4160.5
GLGKAQCAALWLQCASGGTIGCGGGAVACQNYRQFCRNGL
SunA$_{core}$-NGL
Cald. m/z: 4160.9
Found m/z: 4160.5
b
GLGKAQCAALWLQCASGGTIGCGGGAVACQNYRQFCRNGL
4393.5
SunA$_{core}$-NGL + IAA
Cald. m/z: 4393.0
Found m/z: 4393.5
c
3972.4
GLGKAQCAALWLQCASGGTIGCGGGAVACQNYRQFCRN
SunA$_{core}$-NGL + OaAEP1
Cald. m/z: 3972.8
Found m/z: 3972.4
d
4204.7
GLGKAQCAALWLQCASGGTIGCGGGAVACQNYRQFCRN
SunA$_{core}$-NGL
+ OaAEP1, + IAA
Cald. m/z: 4204.9
Found m/z: 4204.7
4000
4200
4400
m/z
4600
4800
e
GLGKAQCAALWLQCASGGTIGCGGGAVACQNYRQFCRNGL
Cam
Cam
Glucose
Cam
Cam
Precursor m/z: 1,099.0085
[M+4H]$^{+4}$
f
GLGKAQCAALWLQCASGGTIGCGGGAVACQNYRQFCRN
Cam
Cam
Glucose
Cam
Cam
Precursor m/z: 1,052.0022
[M+4H-glucose]$^{+4}$
[M+4H]$^{+4}$
m/z

Figure S3. MALDI-TOF MS analysis of (a) His-SUMO-tev-SunA$_{core}$_NGL co-expressed in *E. coli* with the *S*-glycosyl transferase SunS,[12] and treated with TEV protease *in vitro* to remove the SUMO tag; (b) TCEP-reduced, and iodoacetamide (IAA) alkylated peptide from panel a; (c) OaAEP1 treated peptide from panel a resulting in loss of the C-terminal Gly-Leu motif and an additional water from N-to-C cyclization; and (d) TCEP-reduced and IAA-alkylated product of the cyclic peptide resulting from (c). The square brackets connecting Cys residues in the chemical structures represent disulfide bonds. (e) ESI-MS/MS analysis of peptide in panel b; (f) ESI MS/MS analysis of the peptide in panel d. For the cyclized sample, the sugar moiety was labile under our condition for collision induced dissociation. Assignments of b/y ions are shown in Tables S2 and S3. Abbreviations: Cam = carbamidomethyl.

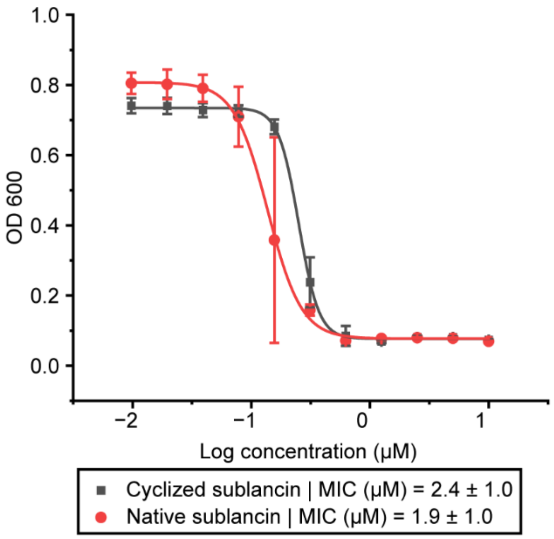

Figure S4. Bioactivity of native sublancin (●) and head-to-tail cyclized sublancin (peptide from panel d, Fig. S3) (■). Bacteria were treated with peptide at the indicated concentrations, grown in LB media at 30 °C and the OD_{600} was measured after 6 h.[15] Indicator strain: *B. subtilis* 168 ΔSPβ.

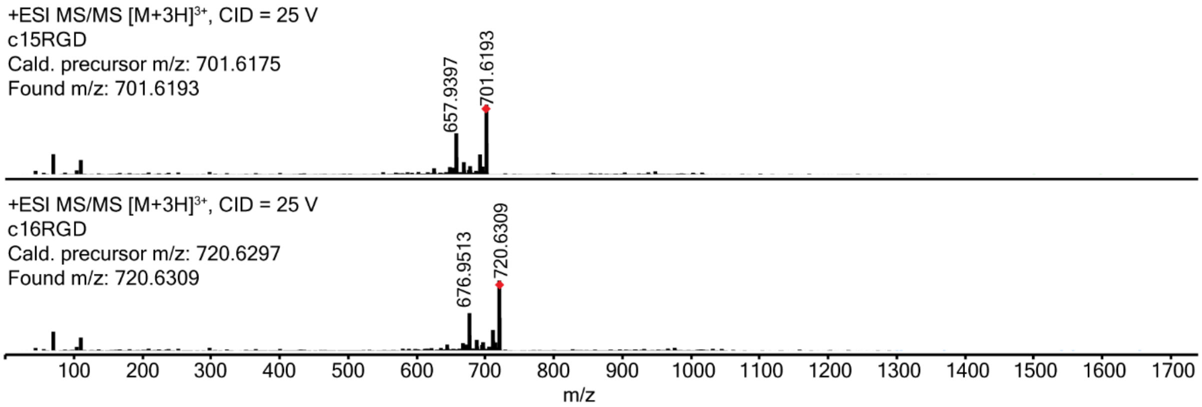

Figure S5. ESI-MS/MS analysis of (a) c15RGD and (b) c16RGD. Since the peptides were head-to-tail cyclized, the MS/MS spectra did not show characteristic b/y ions under similar CID condition that readily fragmented the non-cyclized variants (see Fig. S6). The -44 Da species are likely decarboxylated products of the parent ions.

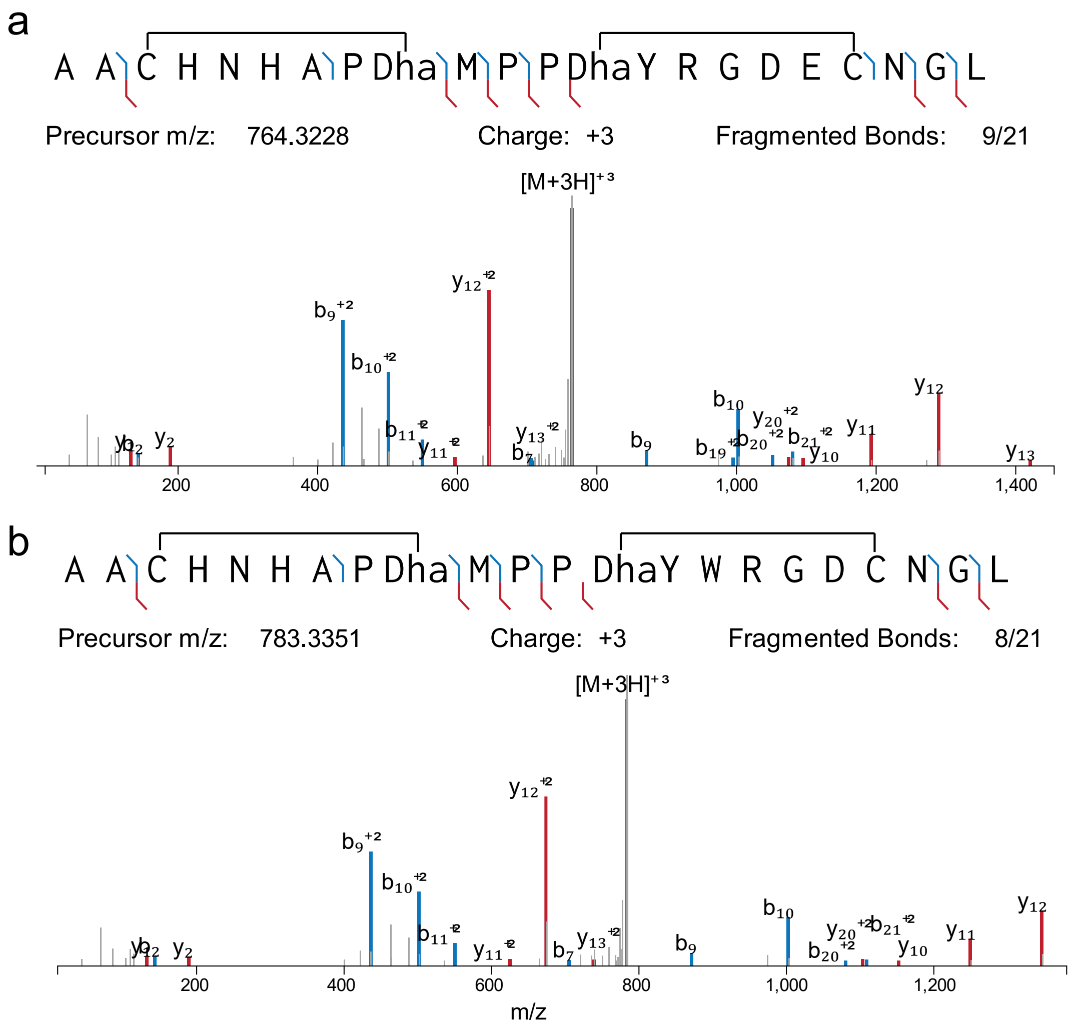

Figure S6. ESI-MS/MS analysis of ProcM-modified (a) 15RGD_NGL and (b) 16RGD_NGL. Matched b/y ions are shown in Tables S4-5.

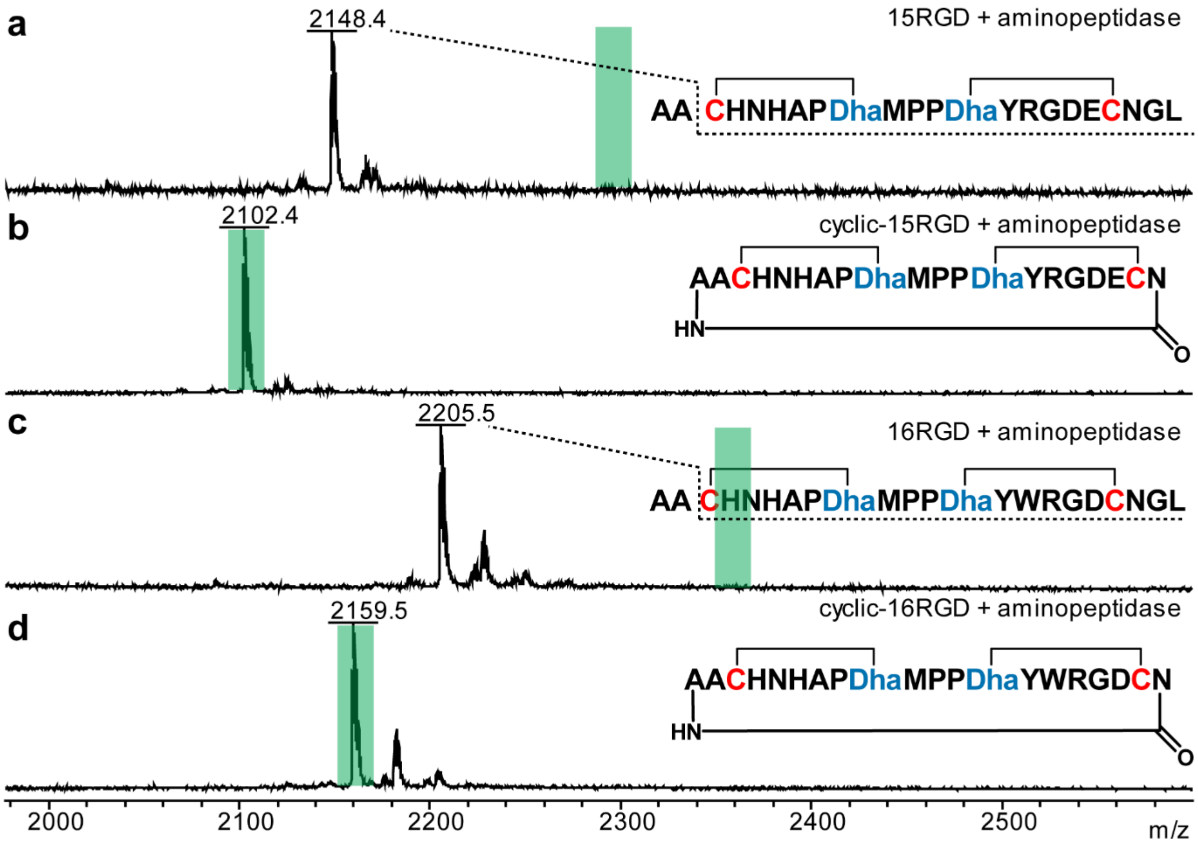

Figure S7. (a-d) Aminopeptidase digestion of 15RGD, c15RGD, 16RGD, and c16RGD. Green boxes indicate the masses of undigested peptides. The dotted line indicates digested fragments resulting from aminopeptidase activity, which was stopped at the lanthionine residues.

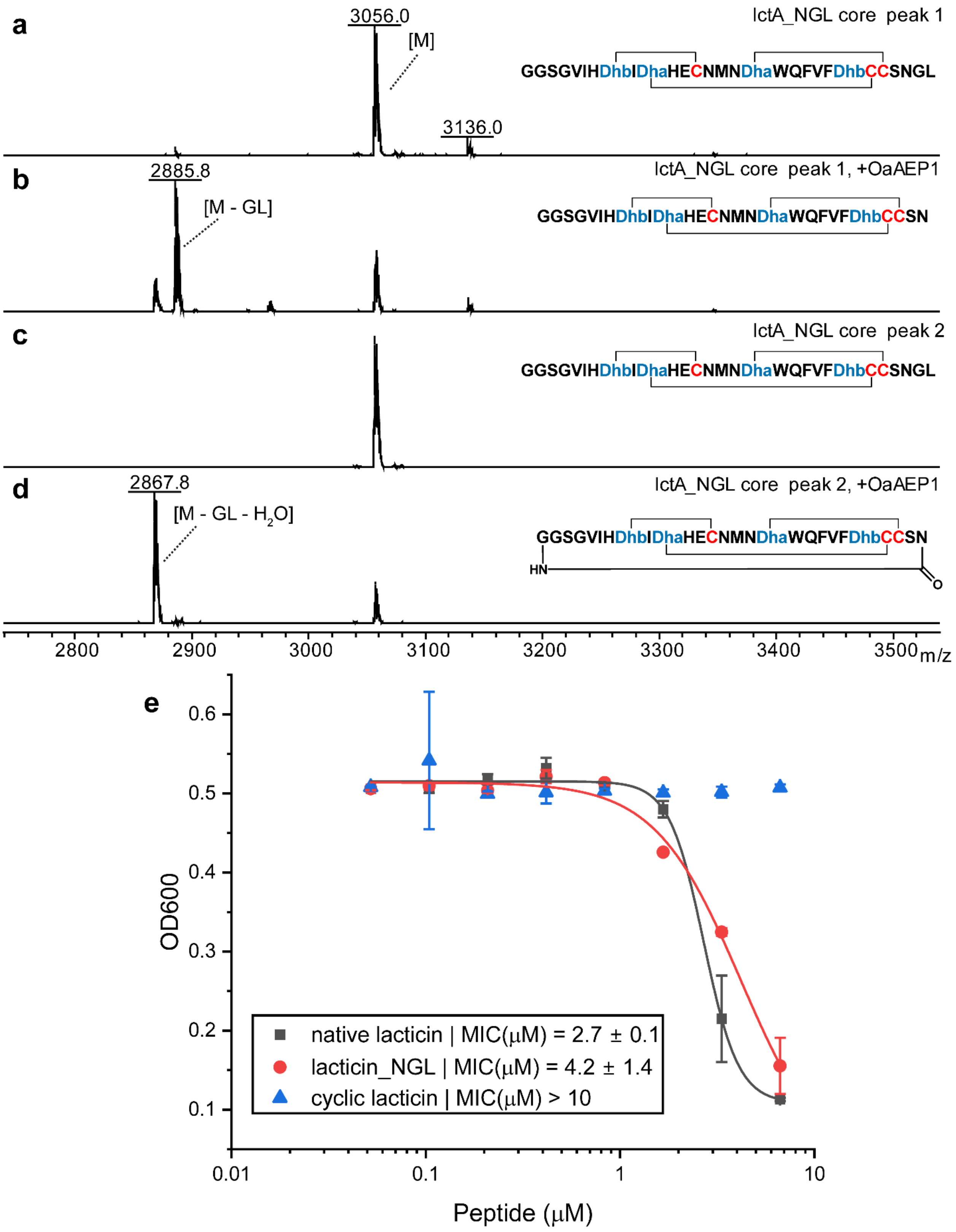

Figure S8. (a,c) HPLC-purified LctA after co-expression with LctM and cleavage by trypsin. (b,d) OaAEP1 treatment of the core peptides. (e) Bioactivity of native lacticin 481 (■), non-cyclized

lctA_NGL core peak 2 (●), and its head-to-tail cyclic product (▲). Bacteria were treated with peptide at the indicated concentrations, grown in GM17 media at 30 °C and the OD_{600} was measured after 18 h. Indicator strain: *L. lactis cremoris.*

Table S1. Assigned b/y ions for Figure S2.

ions	Experimental m/z	Theoretical m/z	Mass Error (ppm)
y1	133.0614	133.0608	4.726
y2	190.083	190.0822	4.0238
y3	376.1621	376.1615	1.475
y4	489.2469	489.2456	2.6466
y10	516.2464	516.2508	-8.5943
y5	546.2669	546.2671	-0.3096
y6	691.3212	691.3198	2.0553
b8	764.3439	764.343	1.2253
y8	861.429	861.4253	4.2915
b10	892.4038	892.4015	2.5308
b20	936.9366	936.9363	0.3587
y9	960.4573	960.4573	0.0175
b11	963.4384	963.4387	-0.2652
M+3H	1140.203	1140.202	1.1217
b26	1230.074	1230.074	0.1707
b14	1278.603	1278.6	2.4093
b29	1364.644	1364.643	0.702
b15	1393.625	1393.627	-1.5495
b30	1437.176	1437.169	5.012
b16	1450.658	1450.649	6.3258
b32	1522.225	1522.222	1.8664
b33	1615.261	1615.261	-0.1325
b18	1660.78	1660.786	-3.1596

Table S2. Assigned b/y ions for Figure S3e.

Ions	Charges	Experimental m/z	Theoretical m/z	Mass Error (ppm)
b2	1	171.1128	171.1128	-0.0027
b4	1	356.2296	356.2292	1.0542
b5	1	427.2677	427.2663	3.1866
b9	2	429.2203	429.2185	4.1044
b10	2	485.7609	485.7606	0.6828
b6	1	555.326	555.3249	1.9476
b11	2	578.8015	578.8002	2.2057
b12	2	635.3445	635.3423	3.5362
b13	2	699.3734	699.3715	2.6548
b7	1	715.3572	715.3556	2.2755

b14	2	779.3876	779.3869	0.9364
b8	1	786.3951	786.3927	3.0695
y12	2	808.3675	808.3645	3.7551
b15	2	814.9079	814.9054	3.0345
y13	2	843.8859	843.883	3.411
b9	1	857.4313	857.4298	1.7492
b16	2	858.4264	858.4214	5.7761
b17	2	886.9343	886.9322	2.3974
y14	2	893.4189	893.4172	1.8709
b18	2	915.4451	915.4429	2.397
y15	2	928.9396	928.9358	4.1063
y26	3	946.0886	946.0892	-0.6358
b19	2	965.9696	965.9667	2.9549
b10	1	970.516	970.5139	2.2048
y17	2	985.9617	985.9572	4.5139
y27	3	999.4378	999.4328	5.0501
y18	2	1014.471	1014.468	2.8768
b20	2	1022.512	1022.509	2.9577
y28	3	1042.123	1042.119	4.082
b21	2	1051.021	1051.02	1.7053
y29	3	1079.82	1079.814	5.8966
M+4H	4	1099.016	1099.009	6.5358
y30	3	1141.845	1141.84	4.6684
y19	2	1147.002	1146.998	3.9002
b11	1	1156.596	1156.593	2.6196
y20	2	1175.514	1175.508	4.544
y21	2	1232.056	1232.05	4.8792
b12	1	1269.679	1269.677	1.3939
y22	2	1282.579	1282.574	3.7202
y23	2	1311.093	1311.085	5.9794
y24	2	1339.603	1339.596	5.5296
y25	2	1383.117	1383.112	3.8992
b13	1	1397.738	1397.736	1.7098
y26	2	1418.636	1418.63	4.3958
y27	2	1498.652	1498.646	4.2766
y28	2	1562.679	1562.675	2.7639
y29	2	1619.221	1619.217	2.7106

Table S3. Assigned b/y ions for Figure S3f.

**Ions**	**Charge**	**Experimental m/z**	**Theoretical m/z**	**Mass Error (ppm)**
y7	1	1043.489	1043.484	4.6336
M+4H	4	1051.986	1052.002	-15.8946
y18	2	1090.515	1090.501	12.7955

b36	3	1306.28	1306.282	-1.9402

Table S4. Assigned b/y ions for Figure S6a.

Ions	Charge	Experimental m/z	Theoretical m/z	Mass Error (ppm)
y1	1	132.1024	132.1019	3.7762
b2	1	143.0815	143.0815	-0.0312
y2	1	189.1236	189.1234	1.2418
b9	2	436.1864	436.185	3.1371
b10	2	501.7072	501.7053	3.8436
b11	2	550.2331	550.2317	2.6287
y11	2	596.7565	596.7562	0.4827
y12	2	645.2836	645.2826	1.5591
b7	1	705.2877	705.2886	-1.2172
y13	2	710.8043	710.8028	2.0625
M+3H	3	764.3237	764.3228	1.1537
b9	1	871.3624	871.3628	-0.4439
b19	2	994.8979	994.9011	-3.1961
b10	1	1002.403	1002.403	-0.2661
b20	2	1051.923	1051.923	-0.0426
y20	2	1074.94	1074.944	-2.8607
b21	2	1080.434	1080.433	0.2992
y10	1	1095.452	1095.452	-0.7152
y11	1	1192.505	1192.505	0.2118
y12	1	1289.56	1289.558	1.542
y13	1	1420.603	1420.598	3.2441

Table S5. Assigned b/y ions for Figure S6b.

Ions	Charge	Experimental m/z	Theoretical m/z	Mass Error (ppm)
y1	1	132.1025	132.1019	4.5332
b2	1	143.0818	143.0815	2.0655
y2	1	189.1239	189.1234	2.828
b9	2	436.1862	436.185	2.6786
b10	2	501.7076	501.7053	4.6409
b11	2	550.2331	550.2317	2.6287
y11	2	625.2769	625.2746	3.7232
y12	2	673.8031	673.801	3.185
b7	1	705.2904	705.2886	2.611
y13	2	739.3217	739.3212	0.6845
M+3H	3	783.3369	783.3351	2.3512
b9	1	871.3618	871.3628	-1.1325
b10	1	1002.405	1002.403	1.2303

b20	2	1080.44	1080.441	-1.0225
y20	2	1103.463	1103.462	0.6025
b21	2	1108.956	1108.952	3.664
y10	1	1152.489	1152.489	-0.003
y11	1	1249.545	1249.542	2.1068
y12	1	1346.6	1346.595	3.764

Protein sequences

> His6_SUMO_OAAEP (C247A)
MGSSHHHHHHSTSDSEVNQEAKPEVKPEVKPETHINLKVSDGSSEIFFKIKKTTPLRRLM
EAFAKRQGKEMDSLRFLYDGIRIQADQTPEDLDMEDNDIIEAHREQIGGSARDGDYLHL
PSEVSRFFRPQETNDDHGEDSVGTRWAVLIAGSKGYANYRHQAGVCHAYQILKRGGLK
DENIVVFMYDDIAYNESNPRPGVIINSPHGSDVYAGVPKDYTGEEVNAKNFLAAILGNK
SAITGGSGKVVDSGPNDHIFIYYTDHGAAGVIGMPSKPYLYADELNDALKKKHASGTYK
SLVFYLEACESGSMFEGILPEDLNIYALTSTNTTESSWAYYCPAQENPPPPEYNVCLGDL
FSVAWLEDSDVQNSWYETLNQQYHHVDKRISHASHATQYGNLKLGEEGLFVYMGSNP
ANDNYTSLDGNALTPSSIVVNQRDADLLHLWEKFRKAPEGSARKEEAQTQIFKAMSHR
VHIDSSIKLIGKLLFGIEKCTEILNAVRPAGQPLVDDWACLRSLVGTFETHCGSLSEYGMR
HTRTIANICNAGISEEQMAEAASQACASIP*
> His6_SboA_NGL
MGSSHHHHHHSQDPNSMKKAVIVENKGCATCSIGAACLVDGPIPDFEIAGATGLFGLW
GNGL*
> His6_SboA(insR2)_NGL
MGSSHHHHHHSQDPNSMKKAVIVENRKGCATCSIGAACLVDGPIPDFEIAGATGLFGLW
GNGL*
>AlbA
MFPFINESVRVHQLPEGGVLEIDYLRDNVSISDFEYLDLNKTAYELCMRMDGQKTAEQI
LAEQCAVYDESPEDHKDWYYDMLNMLQNKQVIQLGNRASRHTITTSGSNEFPMPLHAT
FELTHRCNLKCAHCYLESSPEALGTVSIEQFKKTADMLFDNGVLTCEITGGEIFVHPNAN
EILDYVCKKFKKVAVLTNGTLMRKESLELLKTYKQKIIVGISLDSVNSEVHDSFRGRKGS
FAQTCKTIKLLSDHGIFVRVAMSVFEKNMWEIHDMAQKVRDLGAKAFSYNWVDDFGR
GRDIVHPTKDAEQHRKFMEYEQHVIDEFKDLIPIIPYERKRAANCGAGWKSIVISPFGEV
RPCALFPKEFSLGNIFHDSYESIFNSPLVHKLWQAQAPRFSEHCMKDKCPFSGYCGGCYL
KGLNSNKYHRKKHLLLGKK*
> His-LctA_NGL
MGSSHHHHHHSQDPNSMKEQNSFNLLQEVTESELDLILGAKGGSGVIHTISHECNMNSW
QFVFTCCSNGL*
>LctM
MKKKTYQFEKFLKNTFDQFSIKQNEVLVEDDLNDIIMNVCGKALVLMINEKREMNLLM
GNTPEERYQYFENEYSSTGKAFEEIKDKFPVIYIDLKNSINSYLKLVSQIMKDFKKDYSLL
VERKIIEEHSTISTMKIKGDLHNGKAVIEITTNKSKLIYKPKSLSNDVFFNNFLKYMDSFFI
KEGKSTKYKENFYLVNTLDMKTYGWVEYVDKKPINSFEEARNYYRKIGVLLSVAYTLN
LTDLHFENVISQGENPCIIDLETMFNMPMFVKDYKNESRNIINGKIMDSVVSTGMLPVLG
IDSLFGGDPSGILGGTFSKEERVIINPFRDDIKFQKIVVRSVFKDHIPFFNNNNEKRYCKPK
DYVNDIIKGFEKTYKIIVKNKEKILGFLKKESSSVTCRILFRNTMEYSVLLNAAKSPVYSN
KREEIFEKLSTFNRGLGNDIIKSEISQINTLSIPYFNCQVDSNLIKNMDGETIFEHTLTPFKC
FLSKYRRLCVDDMEQQVKLIRFSIQSQEQLFKDGEQFSLYKKQKGSQEDLLIAINELSSIL
ENNAYIGTSDDTINWMSLGIADNDQILFESLENDIYKGISGIGLALLEYYEFYPNINTKKIL
KLIYKNISKDFINTNNEPQNYGFYVGLIGEYSFLRKYEKVFHKTSSCNILKNILKDFTPEK
CQTILPSDDVIAGEAGIIIYISNLNNYLEYRDEIDILLKSLSNKIKLKESIASYAHGNSGIAT
AFVHGYKVTKNEKYLKIFHELWNLENSSKLRRGWTDSRKVDSSYSSQWCHGASGQAIA
RMEWITVNKTARFLSNSELIKVKKELGELIDILKKEGMYTDNFCLCHGILGNLLILNTYQ

ENFDNKNINLKNEILNNYYSVCNYGLNKGWICGLGTEFYSYGLMTGISGILYGLIRQVK
QKNNFGVLMPYVD*
> His6_SUMO_tev_SunA$_{core}$_NGL (the TEV recognition sequence is underlined)
MPHHHHHHGSSMASMSDSEVNQEAKPEVKPEVKPETHINLKVSDGSSEIFFKIKKTTPLR
RLMEAFAKRQGKEMDSLRFLYDGIRIQADQTPEDLDMEDNDIIEAHREQIGGIEENLYFQ
GLGKAQCAALWLQCASGGTIGCGGGAVACQNYRQFCRNGL*
>SunS
MKLSDIYLELKKGYADSLLYSDLSLLVNIMEYEKDIDVMSIQSLVAGYEKSDTPTITCGII
VYNESKRIKKCLNSVKDDFNEIIVLDSYSTDDTVDIIKCDFPDVEIKYEKWKNDFSYARN
KIIEYATSEWIYFIDADNLYSKENKGKIAKVARVLEFFSIDCVVSPYIEEYTGHLYSDTRR
MFRLNGKVKFHGKVHEEPMNYNHSLPFNFIVNLKVYHNGYNPSENNIKSKTRRNINLTE
EMLRLEPENPKWLFFFGRELHLLDKDEEAIDYLKKSINNYKKFNDQRHFIDALVLLCTLL
LQRNNYVDLTLYLDILETEYPRCVDVDYFRSAILLVDMQNKLTSLSNMIDEALTDERYS
AINTTKDHFKRILISLNIQLENWERVKEISGEIKNDNMKKEIKQYLANSLHNIEHVLKGIE
V*
> His-ProcA2.8(15RGD)_NGL
MGSSHHHHHHSQDPNSMSEEQLKAFLTKVQADTSLQEQLKIEGADVVAIAKAAGFSITT
EDLNSHRQNLSDDELEGVAGGAACHNHAPSMPPSYRGDECNGL*
> His-ProcA2.8(16RGD)_NGL
MGSSHHHHHHSQDPNSMSEEQLKAFLTKVQADTSLQEQLKIEGADVVAIAKAAGFSITT
EDLNSHRQNLSDDELEGVAGGAACHNHAPSMPPSYWRGDCNGL*
>His-LahT150
MGSSHHHHHHSQDPSKKQIQPVTRGRAKVPVIMQMEALECGAASLAMVLAYYKKWVP
LEQVRVDCGVSRDGSNALNVLKAARNYGLEAKGYRYEPEKLKKEGTFPCIIHWNFNHF
VVLKGFKGKYAYINDPAKGDVKIPMEEFDRSFTGICLIFKPTDRFEQ*
>ProcM
MESPSSWKTSWLAAIAPDEPHKFDRRLEWDELSEENFFAALNSEPASLEEDDPCFEEALQ
DALEALKAAWDLPLLPVDNNLNRPFVDVWWPIRCHSAESLRQSFVSDSAGLADEIFDQL
ADSLLDRLCALGDQVLWEAFNKERTPGTMLLAHLGAAGDGSGPPVREHYERFIQSHRR
NGLAPLLKEFPVLGRLIGTVLSLWFQGSVEMLQRICADRTVLQQCFAIPCGHHLKTVKQ
GLSDPHRGGRAVAVLEFADPNSTANSSMHVVYKPKDMAVDAAYQATLADLNTHSDLS
PLRTLAIHNGNGYGYMEHVVHHLCANDKELTNFYFNAGRLTALLHLLGCTDCHHENLI
ACGDQLLLIDTETLLEADLPDHISDASSTTAQPKPSSLQKQFQRSVLRSGLLPQWMFLGE
SKLAIDISALGMSPPNKPERIALGWLGFNSDGMMPGRVSQPVEIPTSLPVGIGEVNPFDRF
LEDFCDGFSMQSEALIKLRNRWLDVNGVLAHFAGLPRRIVLRATRVYFTIQRQQLEPTA
LRSPLAQALKLEQLTRSFLLAESKPLHWPIFAAEVKQMQHLDIPFFTHLIDADALQLGGL
EQELPGFIQTSGLAAAYERLRNLDTDEIAFQLRLIRGAVEARELHTTPESSPTLPPPATPEA
LMSSSAETSLEAAKRIAHRLLELAIRDSQGQVEWLGMDLGADGESFSFGPVGLSLYGGSI
GIAHLLQRLQAQQVSLMDADAIQTAILQPLVGLVDQPSDDGRRRWWRDQPLGLSGCGG
TLLALTLQGEQAMANSLLAAALPRFIEADQQLDLIGGCAGLIGSLVQLGTESALQLALRA
GDHLIAQQNEEGAWSSSSSQPGLLGFSHGTAGYAAALAHLHAFSADERYRTAAAAALA
YERARFNKDAGNWPDYRSIGRDSDSDEPSFMASWCHGAPGIALGRACLWGTALWDEE
CTKEIGIGLQTTAAVSSVSTDHLCCGSLGLMVLLEMLSAGPWPIDNQLRSHCQDVAFQY
RLQALQRCSAEPIKLRCFGTKEGLLVLPGFFTGLSGMGLALLEDDPSRAVVSQLISAGLW
PTE*

References

1. D. R. Brademan, N. M. Riley, N. W. Kwiecien and J. J. Coon, *Mol. Cell. Proteom.*, 2019, **18**, S193-S201.
2. J. D. Hegemann, S. C. Bobeica, M. C. Walker, I. R. Bothwell and W. A. van der Donk, *ACS Synth. Biol*, 2019, **8**, 1204-1214.
3. L. L. Xie, L. M. Miller, C. Chatterjee, O. Averin, N. L. Kelleher and W. A. van der Donk, *Science*, 2004, **303**, 679-681.
4. K. M. Mikula, I. Tascón, J. J. Tommila and H. Iwaï, *FEBS Lett.*, 2017, **591**, 1285-1294.
5. R. Yang, Y. H. Wong, G. K. T. Nguyen, J. P. Tam, J. Lescar and B. Wu, *J. Am. Chem. Soc.*, 2017, **139**, 5351-5358.
6. D. V. Rial and E. A. Ceccarelli, *Protein Expres Purif*, 2002, **25**, 503-507.
7. L. Flühe, T. A. Knappe, M. J. Gattner, A. Schäfer, O. Burghaus, U. Linne and M. A. Marahiel, *Nat. Chem. Biol.*, 2012, **8**, 350-357.
8. B. J. Smithies, Y. H. Huang, M. A. Jackson, K. Yap, E. K. Gilding, K. S. Harris, M. A. Anderson and D. J. Craik, *ACS Chem. Biol.*, 2020, **15**, 962-969.
9. J. I. MacDonald, H. K. Munch, T. Moore and M. B. Francis, *Nat. Chem. Biol.*, 2015, **11**, 326-331.
10. S. C. Bobeica, S. H. Dong, L. Huo, N. Mazo, M. I. McLaughlin, G. Jimenéz-Osés, S. K. Nair and W. A. van der Donk, *eLife*, 2019, **8**, e42305.
11. I. R. Rahman, A. Sanchez, W. Tang and W. A. van der Donk, *ACS Infect. Dis.*, 2021, **7**, 2445-2454.
12. S. Biswas, C. V. Garcia De Gonzalo, L. M. Repka and W. A. van der Donk, *ACS Chem. Biol.*, 2017, **12**, 2965-2969.
13. S. Garneau, N. I. Martin and J. C. Vederas, *Biochimie*, 2002, **84**, 577-592.
14. P. M. Himes, S. E. Allen, S. Hwang and A. A. Bowers, *ACS Chem. Biol.*, 2016, **11**, 1737-1744.
15. C. V. Garcia De Gonzalo, E. L. Denham, R. A. Mars, J. Stulke, W. A. van der Donk and J. M. van Dijl, *Antimicrob. Agents Chemother.*, 2015, **59**, 6844-6854.
